# Spatial mapping of mitochondrial networks and bioenergetics in lung cancer

**DOI:** 10.1038/s41586-023-05793-3

**Published:** 2023-03-15

**Authors:** Mingqi Han, Eric A. Bushong, Mayuko Segawa, Alexandre Tiard, Alex Wong, Morgan R. Brady, Milica Momcilovic, Dane M. Wolf, Ralph Zhang, Anton Petcherski, Matthew Madany, Shili Xu, Jason T. Lee, Masha V. Poyurovsky, Kellen Olszewski, Travis Holloway, Adrian Gomez, Maie St. John, Steven M. Dubinett, Carla M. Koehler, Orian S. Shirihai, Linsey Stiles, Aaron Lisberg, Stefano Soatto, Saman Sadeghi, Mark H. Ellisman, David B. Shackelford

**Affiliations:** 1 Pulmonary and Critical Care Medicine, David Geffen School of Medicine (DGSOM), University of California Los Angeles (UCLA), Los Angeles, CA USA; 2 Department of Neurosciences, University of California San Diego (UCSD), San Diego, CA USA; 3 National Center for Microscopy and Imaging Research, UCSD, San Diego, CA USA; 4 University of Cambridge, Cambridge, UK; 5 Department of Computer Science, UCLA, Los Angeles, CA USA; 6 Department of Computer Science, Yale University, New Haven, CT USA; 7 Imperial College, London, UK; 8 Department of Endocrinology, DGSOM UCLA, Los Angeles, CA USA; 9 Department of Molecular and Medical Pharmacology, UCLA, Los Angeles, CA USA; 10 Crump Institute for Molecular Imaging, UCLA, Los Angeles, CA USA; 11 Jonsson Comprehensive Cancer Center, UCLA, Los Angeles, CA USA; 12 Molecular Imaging Program, Department of Radiology, Stanford University, Stanford, CA USA; 13 Kadmon Corporation, New York, NY USA; 14 Department of Chemistry and Biochemistry, UCLA, Los Angeles, CA USA; 15 Department of Head and Neck Surgery, DGSOM UCLA, Los Angeles, CA USA; 16 Department of Pathology and Laboratory Medicine, DGSOM UCLA, Los Angeles, CA USA; 17 VA Greater Los Angeles Healthcare System, Los Angeles, CA USA; 18 Department of Biological Chemistry, UCLA, Los Angeles, CA USA; 19 Department Hematology and Oncology, DGSOM UCLA, Los Angeles, CA USA; 20 Department of Chemistry and Chemical Biology, McMaster University, Hamilton, Ontario Canada

**Keywords:** Non-small-cell lung cancer, Energy metabolism

## Abstract

Mitochondria are critical to the governance of metabolism and bioenergetics in cancer cells^1^. The mitochondria form highly organized networks, in which their outer and inner membrane structures define their bioenergetic capacity^2,3^. However, in vivo studies delineating the relationship between the structural organization of mitochondrial networks and their bioenergetic activity have been limited. Here we present an in vivo structural and functional analysis of mitochondrial networks and bioenergetic phenotypes in non-small cell lung cancer (NSCLC) using an integrated platform consisting of positron emission tomography imaging, respirometry and three-dimensional scanning block-face electron microscopy. The diverse bioenergetic phenotypes and metabolic dependencies we identified in NSCLC tumours align with distinct structural organization of mitochondrial networks present. Further, we discovered that mitochondrial networks are organized into distinct compartments within tumour cells. In tumours with high rates of oxidative phosphorylation (OXPHOS^HI^) and fatty acid oxidation, we identified peri-droplet mitochondrial networks wherein mitochondria contact and surround lipid droplets. By contrast, we discovered that in tumours with low rates of OXPHOS (OXPHOS^LO^), high glucose flux regulated perinuclear localization of mitochondria, structural remodelling of cristae and mitochondrial respiratory capacity. Our findings suggest that in NSCLC, mitochondrial networks are compartmentalized into distinct subpopulations that govern the bioenergetic capacity of tumours.

## Abstract (web summary)

A study describing an approach that combines imaging and profiling techniques to structurally and functionally analyse lung cancer in vivo, revealing heterogeneous mitochondrial networks and an association between bioenergetic phenotypes and mitochondrial organization and function.

## Main

NSCLC is a heterogeneous disease at a histological, genetic and metabolic level^4^. Mitochondria are essential regulators of cellular energy and metabolism, playing a critical role in sustaining growth and survival of tumour cells^5^. The mitochondria organize into dynamic networks such that the structural architecture of their outer and inner membrane dictates cellular electron transport chain (ETC) activity and respiratory capacity^2,6^. However, our understanding of how mitochondrial networks are structurally and functionally regulated in NSCLC at an in vivo level is limited.

To better understand mitochondrial bioenergetics in NSCLC, we recently developed and validated a voltage-sensitive, positron emission tomography (PET) tracer known as [^18^F]4-fluorobenzyl triphenylphosphonium ([^18^F]FBnTP)^7,8^. This tracer allowed us to measure relative changes in the mitochondrial membrane potential (Δ*Ψ*) in autochthonous KRAS-driven mouse models of NSCLC^9^. PET imaging of NSCLC tumours in KRAS-driven genetically engineered mouse models (GEMMs) identified that lung adenocarcinoma (LUAD) and lung squamous cell carcinoma (LUSC) had distinctly different uptake values for the radiotracers [^18^F]FBnTP and ^18^F[FDG], suggestive of functionally distinct metabolic and bioenergetic phenotypes^9^. Therefore, we sought to determine whether [^18^F]FBnTP uptake in NSCLC tumours directly correlated with OXPHOS activity in vivo.

## In vivo profiling of OXPHOS in NSCLC

**Fig. 1:**
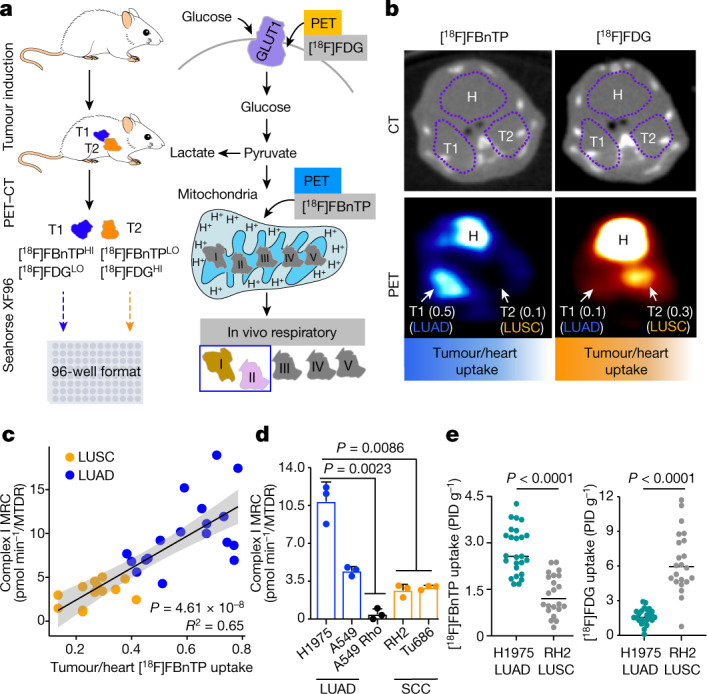
In vivo characterization of mitochondrial bioenergetics and respiration capacity among NSCLC subtypes. **a**, Schematic depicting experimental approach of [^18^F]FBnTP and [^18^F]FDG PET imaging followed by respirometry on frozen tumour samples measuring mitochondrial complex I and complex II MRC. T1, tumour 1; T2, tumour 2. **b**, Representative [^18^F]FBnTP (left) and [^18^F]FDG (right) transverse PET–CT images of KPL mice. Uptake of PET probe was measured as the maximum percentage of injected dose (PID) per gram of tissue. Numbers in brackets after T1 and T2 indicate ratio of uptake tumour to heart. H, heart. **c**, Correlation between the tumour/heart ratio of [^18^F]FBnTP uptake and complex I MRC of tumours from KPL, KL, Kras, KP and LPP mice (*n* = 30 tumours, *n* = 18 LUAD tumours and *n* = 12 LUSC tumours). The MRC values are normalized to mitochondrial content quantified by MitoTracker Deep Red (MTDR). The grey shading represents s.e.m. One-tailed *F*-statistics. **d**, MRC of complex I in frozen xenografts from human cells (H1975, A549, A549 Rho, RH2 and Tu686); data are mean ± s.e.m. (*n* = 3 biological replicates per cell line). One-way analysis of variance (ANOVA), Dunnett test. **e**, [^18^F]FBnTP (*n* = 25 LUAD tumours, *n* = 22 LUSC tumours) and [^18^F]FDG (*n* = 22 LUAD tumours, *n* = 22 LUSC tumours) uptake of xenografts from human NSCLC cell lines (H1975 and RH2). Unpaired two-tailed *t*-test, lines indicate mean value. Source data

**Extended Data Fig. 1:**
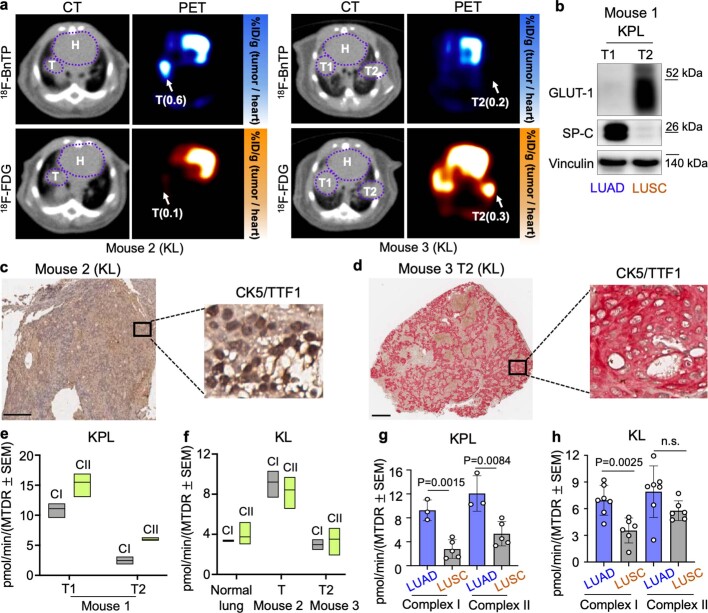
PET/CT imaging, histology and respirometry analysis on *KrasG12D/+ and Lkb1*^*−/−*^ driven tumors. **a**, Representative transverse images of PET/CT probed with ^18^F-BnTP (top) and ^18^F-FDG (bottom) in *KrasG12D/+; Lkb1*^*−/−*^ (KL) mice. Uptake of PET probe was measured by the maximum percentage of inject dose per gram (%ID/g). Ratio of %ID/g by tumor to %ID/g by heart is labeled as %ID/g (tumor/heart). H-heart, T-tumor. **b**, Whole cell lysates of lung tumors T1 and T2 isolated from *KrasG12D/+;* *Lkb1*^*−/−*^; *p53*^*−/−*^ (KPL) mouse were immunoblotted with the antibodies of GLUT1 and SP-C. **c,d**, Immunohistochemical staining of TTF-1 and CK-5 in sections from ^18^F-BnTP^HI^ tumor (mouse 2, left panel) and ^18^F-BnTP^LO^ tumor (T2 in mouse 3, right panel). Scale bar = 500 μm. **e,f**, Maximal respiration capacity (MRC) of mitochondrial Complex I (CI) and Complex II (CII) isolated from frozen tissues of T1 and T2 in mouse 1 (KPL) (**e**) and from T in mouse 2 (KL) and T2 in mouse 3 (KL) (**f**). Data are n = 3 technical replicates, box was interleaved low-high, line at mean. **g,h**, MRC of Complex I and Complex II in LUAD and LUSC from KPL mice (n = 8 tumors, 3 LUAD tumors and 5 LUSC tumors) (**g**) and from KL mice (n = 13 tumors, 7 LUAD tumors and 6 LUSC tumors) (**h**). Data are mean ± s.e.m., unpaired two-tailed t-test. Source data

To evaluate the OXPHOS signatures in NSCLC, we coupled PET imaging of lung tumours using the radiotracers [^18^F]FBnTP and [^18^F]FDG followed by ex vivo respirometry analysis of mitochondrial complex I and II activity (Fig. 1a). [^18^F]FBnTP was used to compare the ratio of mitochondrial Δ*Ψ* in the tumour to that of the heart, whereas [^18^F]FDG was used to measure glucose flux in tumours. Representative [^18^F]FBnTP and [^18^F]FDG PET–CT images of *Kras*^*G12D*^*;p53*^*−/*^^*−*^*;Lkb1*^*−/*^^*−*^ (KPL) and *Kras*^*G12D*^*;Lkb1*^*−/*^^*−*^ (KL) mice identified synchronous lung tumours with differential uptake of the radiotracers (Fig. 1b and Extended Data Fig. 1a), as previously described^9^. Lung tumours with high [^18^F]FBnTP and low [^18^F]FDG uptake were denoted as [^18^F]FBnTP^HI^;[^18^F]FDG^LO^ and glycolytic tumours with the opposite phenotype were termed [18F]FBnTP^LO^;[^18^F]FDG^HI^. In GEMMs, inactivation of *Lkb1* drives the development of both LUAD and LUSC subtypes^10^. Therefore, we confirmed tumour subtypes by staining for surfactant protein C (SP-C) and TTF1 to verify LUAD histology and for glucose transporter 1 (GLUT1) and cytokeratin 5 (CK5) to verify LUSC histology, as previously described^11^ (Extended Data Fig. 1b–d).

The ETC generates a proton gradient to maintain Δ*Ψ* and drive OXPHOS. Therefore, we examined whether uptake of [^18^F]FBnTP correlated with the OXPHOS activity in lung tumours. Following PET imaging, ex vivo respirometry was carried out on snap-frozen lung tumours that allowed for the direct measurement of complex I and II maximal respiratory capacity (MRC)^12^. Here OXPHOS signatures were defined by the combination of [^18^F]FBnTP tracer uptake and complex I and II MRC in tumours. We identified a threefold upregulation in complex I and II MRC in [^18^F]FBnTP^HI^ tumours versus [^18^F]FBnTP^LO^ tumours (Extended Data Fig. 1e,f). Analysis of mitochondrial respiration in LUAD and LUSC tumours from a larger cohort of KPL and KL mice identified a significant increase in complex I and II MRC in LUAD cells versus LUSC cells (Extended Data Fig. 1f,g). Our analysis of mitochondrial activity in KPL and KL GEMMs showed that LUSC cells had significantly lower OXPHOS signatures compared with those of LUAD cells.

**Extended Data Fig. 2:**
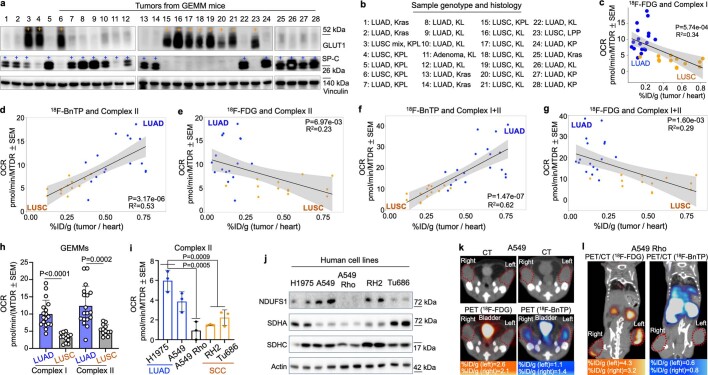
PET/CT imaging, histological markers and respirometry analysis on 5 different genetically modified mouse models (GEMMs) and xenografts of human cells. **a**, Whole cell lysates of lung tumors isolated from KPL, KL, *KrasG12D/+* (Kras), *KrasG12D/+;* *p53*^*−/−*^(KP) and *Lkb1*^*−/−*^; *p53*^*−/−*^; *Pten*^*−/−*^(LPP) mice immunoblotted with the antibodies of GLUT1 and SP-C. **b**, Sample ID, genotype and histology of each tumor are listed. **c**, Correlation between %ID/g (tumor/heart) of ^18^F-FDG uptake and Complex I MRC of tumors from KPL, KL, Kras, KP and LPP mice (n = 30 tumors, n = 18 LUAD tumors and n = 12 LUSC tumors). One-tailed F-statistics. **d–g**, Correlation between %ID/g (tumor/heart) of ^18^F-BnTP (**d,f**) and ^18^F-FDG (**e,g**) uptake and MRC of Complex II (**d,e**) and Complex I+II (**f,g**) in tumors from KPL, KL, Kras, KP and LPP mice (n = 30 tumors, n = 18 LUAD tumors and n = 12 LUSC tumors). One-tailed F-statistics. **h**, MRC of Complex I and Complex II in frozen LUAD cells and LUSC cells from Kras, KL, KP, KPL and LPP mice (n = 30 tumors, 18 LUAD tumors and 12 LUSC tumors. Data are mean ± s.e.m., unpaired two-tailed t-test. **i**, MRC of Complex II in frozen xenografts from human cells (H1975, A549, A549 Rho, RH2 and Tu686); Data are mean ± s.e.m. (n = 3 biological replicates per cell line). One-way ANOVA, Dunnett test. **j**, Immunoblots of Complex I subunit (NDUFS1) and Complex II subunits (SDHA and SDHC) in whole cell lysates from LUAD (H1975, A549, A549 Rho), LUSC (RH2) and HNSCC (Tu686) cell lines. **k**, Transverse images of PET/CT probed with ^18^F-BnTP (right) and ^18^F-FDG (left) in subcutaneous xenografts implanted with A549 cells. Uptake of PET probe was measured by the maximum percentage of inject dose per gram (%ID/g). **l**, Coronal view of PET/CT overlayed images probed with ^18^F-BnTP (right) and ^18^F-FDG (left) in subcutaneous xenografts implanted with A549 Rho cells. Uptake of PET probe was measured by the maximum percentage of inject dose per gram (%ID/g). Source data

Next, we broadened our correlative examination of complex I and II MRC and [^18^F]FBnTP uptake in GEMMs across five different genetic backgrounds: KPL and KL, as well as *Kras*^*G12D*^*;p53*^*−/*^^*−*^ (KP), *Kras*^*G12D*^ (Kras) and *Lkb1*^*−/*^^*−*^*;p53*^*−/*^^*−*^*;Pten*^*−/*^^*−*^ (LPP) mice (Extended Data Fig. 2a,b). We identified a direct and significant correlation between [^18^F]FBnTP uptake and complex I and II MRC (Fig. 1c and Extended Data Fig. 2d,f). Clear histologic stratification was observed, as LUAD cells had higher [^18^F]FBnTP uptake and complex I and II MRC compared with those of LUSC cells (Fig. 1c and Extended Data Fig. 2d,f,h). Conversely, [^18^F]FDG uptake was inversely correlated with complex I and II MRC (Extended Data Fig. 2c,e,g).

We then evaluated [^18^F]FBnTP uptake and complex I and II MRC in human LUAD and squamous cell carcinoma (SCC) cell lines to determine whether OXPHOS signatures were conserved across species. The human LUAD cell lines (H1975 and A549), LUSC cell line (RH2), and head and neck squamous cell carcinoma (HNSCC) cell line (Tu686) were implanted into mouse tumour xenografts. H1975 and A549 tumours had significantly higher complex I and II MRC than tumours from RH2, Tu686 or A549 Rho cells (Fig. 1d and Extended Data Fig. 2i). We also confirmed that A549 Rho cells lacked expression of ETC proteins and uptake of [^18^F]FBnTP (Extended Data Fig. 2j–l). PET imaging of xenografts showed a significantly higher [^18^F]FBnTP uptake in H1975 tumours versus RH2 tumours (Fig. 1e, left). Conversely, RH2 tumours had significantly higher [^18^F]FDG uptake than H1975 tumours (Fig. 1e, right). Collectively, our results demonstrated that [^18^F]FBnTP uptake directly correlated with complex I and II activity in both human and mouse NSCLC. Although LUAD cells tended to have higher OXPHOS signatures than LUSC, given the heightened metabolic heterogeneity that is common to human NSCLC^4^, we anticipate that OXPHOS signatures may vary between NSCLC tumour subtypes.

## PET-guided 3D SBEM imaging of NSCLC

**Fig. 2:**
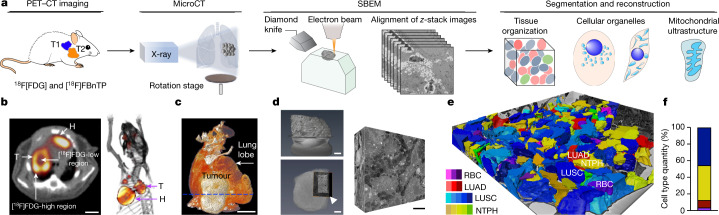
PET-guided multi-modality imaging to characterize spatial architecture of mitochondrial networks in NSCLC. **a**, Schematic of multi-modality imaging technique and analysis approach. **b**, [^18^F]FDG transverse (left) and 3D-rendered (right) PET–CT images. [^18^F]FDG^HI^ tumour (KL) was identified with heterogeneous regions of high and low [^18^F]FDG uptake. Scale bar, 5 mm. **c**, 3D-rendered microCT image of isolated lung lobe with tumour in **b**. Dashed line represents the orientation of sectioning plane on the tumour. Scale bar, 5 mm. **d**, Left: high-resolution microCT images on heavy-metal-stained tumour block. Selected region for SBEM imaging is indicated by white arrowhead. Scale bars, 500 μm (top) and 1 mm (bottom). Right: representative subvolume of SBEM images. Scale bar, 10 μm. **e**, The landscape of SBEM-imaged OXPHOS^LO^ LUSC tumour volume after individual cell segmentation and cell-type classification. LUSC, blue; neutrophil (NTPH), yellow; red blood cell (RBC), purple; LUAD, red. **f**, Quantification of different cell types in **e**. Source data

**Extended Data Fig. 3:**
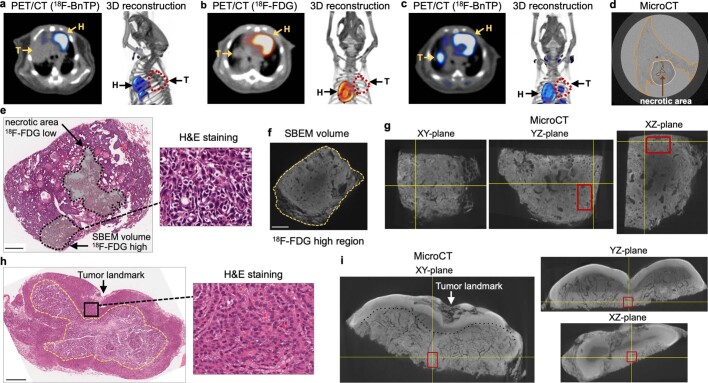
PET guided microCT analysis to select regions for SBEM imaging. **a,b,c**, Transverse view and 3D reconstruction of PET/CT overlayed images probed with ^18^F-BnTP (**a,c**) and ^18^F-FDG (**b**) of ^18^F-FDG^HI^ LUSC tumor in Fig. 2b (**a**) and ^18^F-BnTP^HI^ LUAD tumor (**b,c**). **d**, MicroCT image showed the position of tumor (white outlined) in the lung lobe (orange outlined). Dense tumor region is distinguished from tissue sparse necrotic area (brown outlined) by tissue density. **e**,**h**, Hematoxylin and eosin (H&E) staining of sections from OXPHOS^LO^ LUSC tumor (**e**) and OXPHOS^HI^ LUAD tumor (**h**). Scale bar = 500 μm. (**e**) Arrows indicated necrotic area and selected region for SBEM imaging in OXPHOS^LO^ LUSC tumor. (**h**) Arrow indicated the tumor landmark, box indicated selected region for SBEM imaging in OXPHOS^HI^ LUAD tumor. **f**, High-resolution microCT image of selected SBEM region with high ^18^F-FDG signal and dense tumor tissue indicated in (**e**). Scale bar = 200 μm. **g**,**i**, Cross-sections of XY, YZ and XZ planes in 3D rendered microCT images on heavy-metal stained OXPHOS^LO^ LUSC tumor (**g**) and OXPHOS^HI^ LUAD tumor (**i**). Selected regions for SBEM imaging were indicated in the red boxes.

The fact that we observed functionally distinct OXPHOS signatures in NSCLC tumour subtypes suggests that these tumours may have equally distinct structural organization of their mitochondrial networks. To investigate this, we developed a workflow that paired functional PET imaging with micro-computed tomography (microCT) and ultra-resolution three-dimensional serial block-face electron microscopy^13^ (3D SBEM; Fig. 2a). The incorporation of microCT imaging allowed us to bridge gaps in resolution scales between whole-tumour imaging with PET and ultrastructure imaging with SBEM. We first carried out [^18^F]FBnTP and [^18^F]FDG PET–CT imaging on KRAS(G12D)-driven GEMMs to distinguish mitochondrial activity and glucose flux in tumours (Fig. 2b). We identified regions of high versus low radiotracer uptake within each tumour that were used to guide SBEM analysis (Fig. 2b and Extended Data Fig. 3a–c). We then carried out microCT and histological analysis to: provide an overview of the tumour tissue density from the periphery to core regions; spatially orientate tumours in an *x*–*y*–*z* plane; and differentiate viable versus necrotic tissue (Fig. 2c, Extended Data Fig. 3d,e,h and Supplementary Video 1).

With this workflow in place, we next carried out high-resolution microCT scans on tumours to verify that the tumour sections had proper depth of penetration of heavy metal staining and contained viable, tumour-dense tissue (Fig. 2d, Extended Data Fig. 3f,g,i and Supplementary Video 2). Last, sequential SBEM images were acquired and compiled to generate 3D tumour volumes (Fig. 2d and Supplementary Videos 3–5). SBEM imaging readily facilitates quantitative analysis on large-volume content-rich datasets based on the following three features: tomography (serial sectioning) and 3D rendering of tissue; a large field of view with content-rich images; and nanometre resolution.

**Extended Data Fig. 4:**
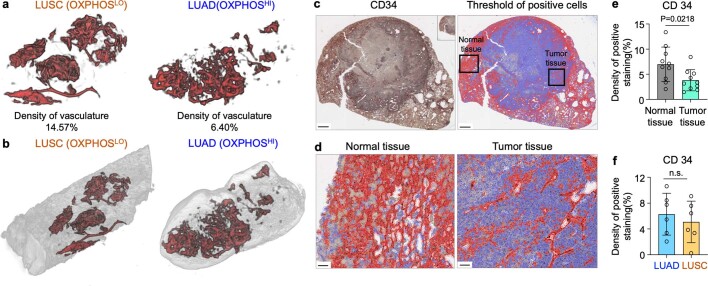
Vascular density in lung normal tissues and tumor tissues measured by microCT and endothelial marker. **a,b**, Reconstruction of vascular structure (**a**) segmented from microCT images (**b**) by gaussian and binary morphological filters in OXPHOS^LO^ LUSC (left panel) and OXPHOS^HI^ LUAD (right panel) tumors. The density of vasculature was indicated. **c,d**, Representative of immunohistochemical staining of CD 34 on the section of a OXPHOS^LO^ LUSC tumor (**c**, left panel). The threshold of positive CD 34 staining was identified using QuPath and indicated by red labeling (**c**, right panel). Scale bar (**c**) = 500 μm. Selected normal tissue region and tumor region in black boxes were zoomed-in (**d**). Scale bar (**d**) = 50 μm. **e,f**, Density of positive CD 34 staining in normal tissues (n = 10) and tumor tissues (n = 10) (**e**), and in LUAD (n = 6) and LUSC (n = 6) (**f**). Data are mean ± s.e.m., unpaired two-tailed t-test. Source data

The reduced [^18^F]FBnTP uptake in LUSC raised the question as to whether these tumours had reduced vasculature as compared to LUAD. We quantified tumour volume and vasculature using microCT analysis and immunohistochemical staining for the endothelial marker CD34. The data showed no notable differences in vasculature densities (Extended Data Fig. 4a,b), or CD34 staining (Extended Data Fig. 4c,f), between OXPHOS^HI^ LUAD and OXPHOS^LO^ LUSC. By contrast, CD34 staining was significantly increased in normal tissue versus tumour (Extended Data Fig. 4d,e).

**Extended Data Fig. 5:**
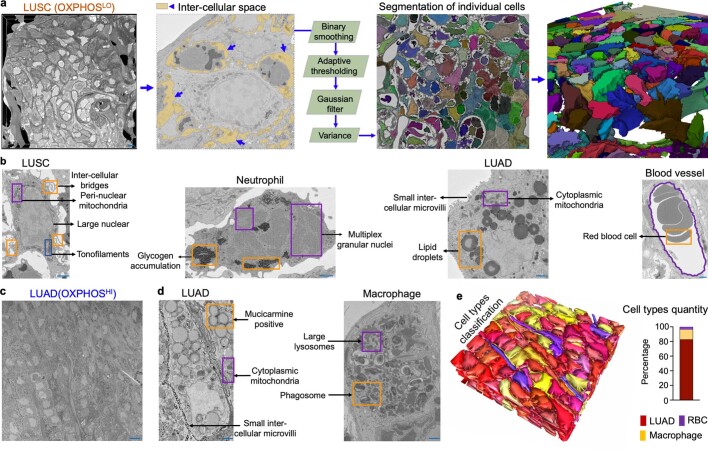
Individual cell segmentation and cell type identification in LUSC and LUAD SBEM volumes. **a**, SBEM volume (75 μm*75 μm*12 μm) of OXPHOS^LO^ LUSC tumor (left panel). Inter-cellular space between LUSC cells and neutrophils is colored as yellow and images were processed in Amira (central panel) with steps of binary smoothing, adaptive thresholding, Gaussian filter and variance to achieve individual cell segmentation. Individual cells were segmented from serial 2D SBEM images were reconstructed in 3D volume (right panel). Scale bar = 10 μm. **b**, Morphological features and special organelle structures were used to distinguish the cell types of LUSC, neutrophil (NTPH), LUAD and red blood cell (RBC) in OXPHOS^LO^ LUSC SBEM volume. Scale bar = 3 μm. **c**, Representative 2D SBEM image of OXPHOS^HI^ LUAD tumor. Scale bar = 15 μm. **d**, Morphological features and special organelle structures identified in the cell types of LUAD and macrophage from OXPHOS^HI^ LUAD SBEM images. Scale bar = 3 μm. **e**, The landscape of SBEM imaged OXPHOS^HI^ LUAD tumor volume after individual cell segmentation and cell-type classification (left panel). Quantification of different cell types (right panel). LUAD-red, macrophage-yellow, red blood cell (RBC)-purple. Source data

Having generated a 3D tumour volume, we next built a topographical map of the tumour’s cellular landscape to identify specific cell types within the tumour. We carried out cell segmentation and labelling of each cell type (tumour versus immune versus endothelial) based on cellular morphological features (Fig. 2e, Extended Data Fig. 5a and Supplementary Video 6). In LUSC, the main immune cell types identified were neutrophils, whereas macrophages were most predominant in LUAD (Fig. 2e,f and Extended Data Fig. 5b–e). These results are consistent with those of previous work in mouse models and human NSCLC^14,15^, and demonstrate the development of an in vivo imaging workflow that enabled us to bridge non-invasive PET imaging of whole tumours with high-resolution microCT and ultra-resolution SBEM.

## Spatial and structural mapping of mitochondria

**Fig. 3:**
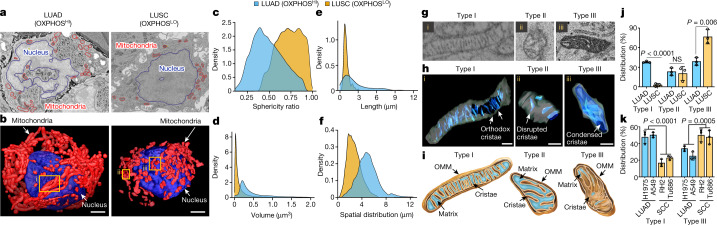
Structural and spatial analysis of mitochondrial networks in SBEM-imaged NSCLC tumour volumes. **a**, Representative 2D SBEM images of an OXPHOS^HI^ LUAD cell and an OXPHOS^LO^ LUSC cell. **b**, 3D reconstruction of nucleus (blue) and mitochondrial (red) networks segmented from NSCLC cells in **a**. Yellow boxes show elongated mitochondria (i) and fragmented mitochondria (ii,iii). Scale bars, 3 μm. **c**–**f**, Density plots measuring mitochondrial sphericity (**c**), volume (**d**), length (**e**) and spatial distribution relative to nucleus surface (**f**) for OXPHOS^HI^ LUAD cells (*n* > 50,000 mitochondria) and OXPHOS^LO^ LUSC cells (*n* > 22,000 mitochondria). **g**,**h**, SBEM images and 3D reconstruction of representative type I (i), II (ii) and III (iii) crista structures identified in OXPHOS^HI^ LUAD cells and OXPHOS^LO^ LUSC cells. Scale bars, 500 nm (**h**). **i**, Illustration of type I, II and III crista structures. Outer mitochondrial membrane (OMM), matrix and inner mitochondrial membrane (cristae) are indicated. **j**, Percentage of mitochondrial type I, II and III crista distribution in OXPHOS^HI^ LUAD cells (*n* = 3 biological replicates, *n* > 1,200 mitochondria) and OXPHOS^LO^ LUSC cells (*n* = 3 biological replicates, *n* > 750 mitochondria). Data are mean ± s.e.m. Unpaired two-tailed *t*-test. **k**, Percentage of type I and III crista distribution in human LUAD (H1975, A549) and SCC (RH2, Tu686) cells. Data are mean ± s.e.m. (*n* = 3 biological replicates, *n* > 2,000 mitochondria). Unpaired two-tailed *t*-test. Source data

Having obtained SBEM datasets from OXPHOS^HI^ LUAD and OXPHOS^LO^ LUSC tumours, we carried out a quantitative structural and spatial analysis of mitochondrial networks within tumour cells. Mitochondria in 2D SBEM images appeared phenotypically similar in both LUAD and LUSC (Fig. 3a). These images accurately represented cross-sectional views of the mitochondria; however, they did not characterize the higher-order organization of mitochondrial networks. By contrast, 3D renderings of mitochondrial networks (shown in red) identified evident phenotypic differences in which LUAD cells had predominantly fused, elongated mitochondria whereas LUSC cells had predominantly fragmented mitochondria (Fig. 3b). These results demonstrated that qualitatively, 3D rendering of tumours identified differences in mitochondrial structure and spatial distribution between LUAD and LUSC cells.

**Extended Data Fig. 6:**
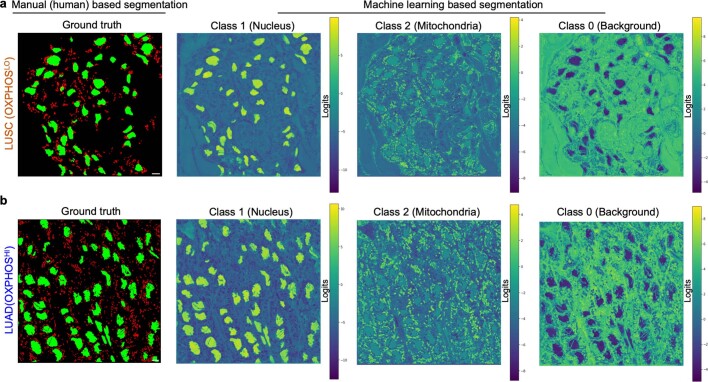
Machine learning based trinary segmentation of nucleus, mitochondria and background in SBEM images. **a,b**, manual labeling of nucleus (green) and mitochondria (red) was used as ground truth (left panel). U-Net encoder decoder architecture of convolution neural network (CNN) was trained for the trinary segmentation of class 1 (nucleus), class 2 (mitochondria) and class 3 (background) in OXPHOS^HI^ LUAD and OXPHOS^LO^ LUSC SBEM volumes (right panel). Scale bar = 6 μm.

Next we developed quantitative methods to analyse mitochondrial structure across our content-rich SBEM datasets. To achieve a comprehensive analysis of the large mitochondrial content within the SBEM volumes, we developed a deep learning convolutional neural network (CNN). The CNN achieved robust and accurate trinary segmentation that included partitioning of the image space of mitochondria, nucleus and background classes. We segmented the mitochondria and nuclei in about 200 LUAD and about 150 LUSC cells from each tumour volume and manually annotated nuclei and mitochondria in 13 2D SBEM images—10 for training and 3 for validation. Our network outputs class logits at each pixel, with the class yielding the highest logits denoting its label. The resulting segmentation yielded an average precision of 0.916 and a recall of 0.927 versus manual registration (Extended Data Fig. 6a,b). We used the CNN to infer the segmentation of 200–500 serial 2D SBEM images to generate 3D renderings of mitochondria and nuclei in lung tumours.

**Extended Data Fig. 7:**
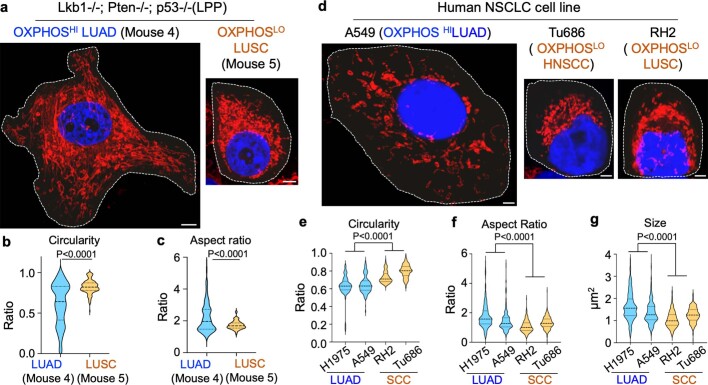
Morphological analysis of mitochondrial networks in mouse and human NSCLC cells cultured *in vitro*. **a**, Representative confocal Airyscan images stained with Mitotracker deep red (MTDR, red) and Hoechst (blue) of mouse NSCLC cells derived from OXPHOS^HI^ LUAD (mouse 4, LPP) and OXPHOS^LO^ LUSC (mouse 5, LPP). Scale bar = 5 μm. **b,c**, Violin plots showing mitochondrial morphological descriptors (circularity and aspect ratio) in mouse LUAD and LUSC cells, n > 150 cells per cell line, 3 biological replicates; unpaired two-tailed t-test. **d**, Representative confocal Airyscan images stained with MTDR (red) and Hoechst (blue) of human OXPHOS^HI^ LUAD (H1975, A549) and OXPHOS^LO^ SCC (RH2, Tu686) cell lines. Scale bar = 4 μm. **e–g**, Violin plots of mitochondrial morphological descriptors (circularity and aspect ratio) and mitochondrial size (area) in human LUAD cell lines (H1975, A549) and SCC cell lines (RH2, Tu686). Data are from n = 3 biological replicates, n > 240 cells per cell line, unpaired two-tailed t-test. Source data

Mitochondria are dynamic organelles that continually remodel their networks resulting in pools that vary in both size and shape. Utilizing our deep learning CNN, we then analysed and quantified 20,000–50,000 mitochondria per tumour section with morphological measurements taken for length, total volume, sphericity (roundness) and spatial distribution in our SBEM tumour volumes. Mitochondria in OXPHOS^LO^ LUSC were predominantly fragmented with increased sphericity versus OXPHOS^HI^ LUAD cells (Fig. 3b,c). Density plots measuring total mitochondrial length and volume showed a narrow distribution in LUSC versus a broad Gaussian distribution of peaks in LUAD cells (Fig. 3d,f and Extended Data Fig. 7a–c). These results showed that mitochondria within OXPHOS^LO^ LUSC cells were smaller and more fragmented than OXPHOS^HI^ LUAD cells.

We followed these results with an analysis of mitochondrial dynamics in human tumour cell lines and found that the OXPHOS^LO^ RH2 and Tu686 SCC lines had significantly higher mitochondrial fragmentation (as measured by mitochondrial circularity, aspect ratio and size) than OXPHOS^HI^ H1975 and A549 LUAD cell lines (Extended Data Fig. 7d–g). Our analysis of human and mouse tumour cells identified a more uniform organization of mitochondrial dynamics in OXPHOS^LO^ SCC cells than in OXPHOS^HI^ LUAD cells.

**Extended Data Fig. 8:**
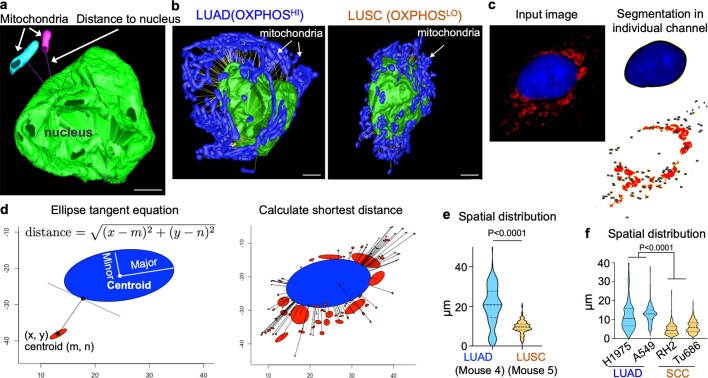
Spatial analysis of mitochondrial networks in mouse and human NSCLC cells cultured *in vitro*. **a**, Illustration of the distance between nucleus and mitochondrial meshed surface using mtk program in Imod. Scale bar = 5 μm. **b**, Mitochondrial spatial distribution was relative to nucleus and measured by the distance between individual mitochondria to the surface of corresponding nucleus in OXPHOS^HI^ LUAD (left panel) and OXPHOS^LO^ LUSC (right panel) cells imaged by SBEM. Scale bar = 3 μm. **c,d**, Schematic of the method developed for measuring the distance between nucleus and mitochondria in 2D confocal Airyscan images. Nucleus and mitochondria are segmented in ImageJ and reconstructed in ellipse shape with the parameters of centroid coordinates, major and minor axes, and angle. The shortest distance between mitochondrial centroid and nucleus ellipse equation is estimated by solving the Lagrangian function. **e,f**, Violin plots of the spatial distribution of mitochondria network in mouse (**e**) and human (**f**) OXPHOS^HI^ LUAD and OXPHOS^LO^ LUSC/SCC cells. Data are from n = 3 biological replicates, n > 150 cells per cell line (**e**), n > 240 cells per cell line (**f**), unpaired two-tailed t-test. Source data

Moreover, we observed that mouse LUAD and LUSC had different spatial distribution of their mitochondria throughout the cell (Fig. 3b). To further evaluate, we developed another algorithm to measure the spatial distance between individual mitochondria and their corresponding nucleus in both SBEM and fluorescent images (Extended Data Fig. 8a–d). In SBEM images from OXPHOS^HI^ LUAD cells, we discovered a broad spatial distribution of mitochondrial networks across the cytoplasm—spanning from the nucleus to the plasma membrane. By contrast, the mitochondria in OXPHOS^LO^ LUSC were enriched at perinuclear regions of the cell (Fig. 3f, Extended Data Fig. 8e and Supplementary Videos 7 and 8). Next we analysed the spatial distribution of mitochondria relative to the nucleus in human tumour cell lines and found that mitochondrial networks in OXPHOS^HI^ LUAD A549 and H1975 cells were localized predominantly throughout the cytoplasm, whereas mitochondrial localization was perinuclear in OXPHOS^LO^ squamous RH2 and Tu686 cells (Extended Data Fig. 8f). These results indicate that OXPHOS^LO^ SCC cells maintained a more spatially restricted and structurally homogeneous pool of fragmented perinuclear mitochondria (PNM) than OXPHOS^HI^ LUAD cells.

**Extended Data Fig. 9:**
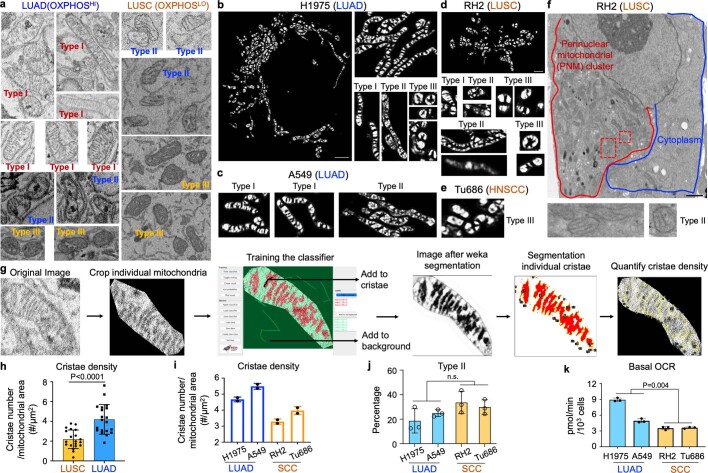
Mitochondrial cristae types in mouse and human NSCLC cells imaged by SBEM and confocal Airyscan. **a**, Representative of mitochondrial cristae structure in SBEM images of OXPHOS^HI^ LUAD (left panel) and OXPHOS^LO^ LUSC (right panel). The classification of type I, II and III cristae was indicated in the representative images. **b–e**, Representative of mitochondrial cristae structure of OXPHOS^HI^ LUAD (**b,c**) and OXPHOS^LO^ LUSC (**d,e**) cells imaged by confocal Airyscan and processed by WEKA segmentation (ImageJ). The classification of type I, II and III cristae was indicated in the representative images. Scale bar (**b**) = 3 μm. Scale bar (**d**) = 2 μm. **f**, Transmitted electron microscopy (TEM) image of OXPHOS^LO^ LUSC (RH2) cell. Zoomed-in images from red boxes illustrated type II cristae structure. Scale bar = 3 μm. **g**, Workflow of mitochondrial cristae segmentation and quantification in SBEM images by trainable WEKA segmentation (ImageJ). **h**, Mitochondrial cristae density (cristae number/mitochondrial area) in OXPHOS^HI^ LUAD and OXPHOS^LO^ LUSC cells imaged by SBEM (n = 20 mitochondria per tumor type). Data are mean ± s.e.m., unpaired two-tailed t-test. **i**, Mitochondrial cristae density (cristae number/mitochondrial area) in human OXPHOS^HI^ LUAD (H1975, A549) and OXPHOS^LO^ SCC (RH2, Tu686) cells. Data are mean ± s.e.m. (n = 2 biological replicates, >100 cells per cell line). **j**, Percentage of type II cristae distribution in human LUAD (H1975, A549) and SCC (RH2, Tu686) cells. Data are mean ± s.e.m. (n = 3 biological replicates, *n* > 2,000 mitochondria). Unpaired two-tailed t-test. **k**, Basal OCR of in human OXPHOS^HI^ LUAD (H1975, A549) and OXPHOS^LO^ SCC (RH2, Tu686) cells. Data are mean ± s.e.m. (n = 3 biological replicates). Unpaired two-tailed t-test. Source data

The SBEM imaging at a resolution of 5–6 nm enabled us to investigate not only mitochondrial dynamics but also the organization of the ultrastructure of the cristae, which constitute the mitochondrial inner membrane. It is well established that the structural organization of the cristae directly impacts the bioenergetic capacity of the mitochondria^2,3,6^. The differences in OXPHOS signatures between NSCLC tumour subtypes suggested that OXPHOS^HI^ LUAD cells may have distinctly different crista organization from OXPHOS^LO^ LUSC cells. Among the OXPHOS^HI^ LUAD and OXPHOS^LO^ LUSC tumour volumes analysed, we identified three main crista structures that have been previously described in mammalian cells. These structures include: highly organized orthodox or lamellar cristae that we classified as type I (ref. ^16^); sparse and disorganized cristae that we classified as type II (ref. ^17^); and condensed cristae that we classified at type III (ref. ^18^). Representative images of type I–III cristae in mouse and human NSCLC cells are shown in Fig. 3g–i and Extended Data Fig. 9a–f.

Orthodox, lamellar cristae support robust OXPHOS activity in cells, whereas disorganized and condensed cristae are associated with defects in cellular OXPHOS activity^16,18–20^. We therefore analysed and compared crista density in mouse and human OXPHOS^HI^ LUAD versus OXPHOS^LO^ LUSC cells. The crista density was quantified using a Weka segmentation (ImageJ) program^21^ (Extended Data Fig. 9g). Morphological analysis identified that LUAD cells had higher crista density and basal oxygen consumption rate (OCR) than LUSC cells (Extended Data Fig. 9h,i). Next we carried out an analysis of crista architecture in human and mouse OXPHOS^HI^ LUAD and OXPHOS^LO^ SCC cells. Quantification of crista types showed that OXPHOS^HI^ LUAD cells had a mixed population of type I, II and III cristae. By contrast, OXPHOS^LO^ SCC cells were enriched for condensed type III cristae with a significant reduction in type I cristae (Fig. 3j,k and Extended Data Fig. 9j). This corresponded to significantly lower basal OCR in squamous versus LUAD cells (Extended Data Fig. 9k). In sum, our structural analysis of mitochondria showed that human and mouse OXPHOS^LO^ tumour cells consistently lacked organized type I cristae compared to OXPHOS^HI^ tumour cells.

## Peri-droplet mitochondria enriched in LUAD

**Fig. 4:**
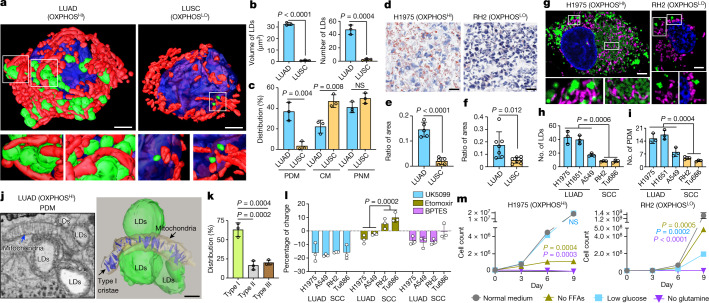
Enrichment of LDs and PDM in OXPHOS^HI^ LUAD cells. Data are mean ± s.e.m. (*n* = 3 biological replicates), unpaired two-tailed *t*-test unless specified otherwise. **a**, 3D reconstruction of LDs (green), mitochondria (red) and nucleus (blue) in an OXPHOS^HI^ LUAD cell and an OXPHOS^LO^ LUSC cell. Zoomed-in images (lower panels) are of the regions outlined in white from the  3D reconstructed cells (upper panels). The lower left panel is a side view of the interaction between mitochondria and LDs. The lower right panel comprises a front and back view of LDs in close proximity to but not contacting mitochondria. Scale bars, 3 μm. **b**, Quantification of the total volume and number of LDs in 3D-rendered LUAD and LUSC cells imaged by SBEM (*n* > 150 LDs). **c**, Percentage of spatially compartmentalized mitochondria in OXPHOS^HI^ LUAD cells (*n* = 3 biological replicates, *n* > 1,200 mitochondria) and OXPHOS^LO^ LUSC cells (*n* = 3 biological replicates, *n* > 750 mitochondria). **d**, Co-staining of oil red O and haematoxylin in OXPHOS^HI^ LUAD (H1975) and OXPHOS^LO^ LUSC (RH2) human xenografts. Scale bars, 40 μm. **e**,**f**, Ratio of area between oil red O and haematoxylin staining for OXPHOS^HI^ LUAD and OXPHOS^LO^ LUSC xenografts (**e**, *n* = 5 LUAD tumours, *n* = 5 LUSC tumours) and GEMMs (**f**, *n* = 6 LUAD tumours, *n* = 7 LUSC tumours). **g**, Co-staining of MTDR (purple), BODIPY (green) and Hoechst (blue) in H1975 and RH2 cells. Scale bars, 3 μm. **h**,**i**, Average number of LDs and PDM in human LUAD and SCC cells (*n* > 300 cells per cell line). **j**, 2D SBEM image (left) and 3D reconstruction (right) of PDM and associated crista structure of an OXPHOS^HI^ LUAD cell. Scale bar, 500 nm. **k**, Percentage of type I, II and III cristae in PDM population (*n* > 400 mitochondria). One-way ANOVA, Dunnett test. **l**, Percentage of change in basal OCR of human LUAD and SCC cells in response to UK5099, etomoxir and BPTES. **m**, Cell count of H1975 and RH2 cells proliferating under the conditions of normal medium (25 mM glucose), and medium with no free fatty acids (FFAs), low glucose (12 mM) or no glutamine. Source data

SBEM imaging of content-rich tumour volumes allowed for 3D mapping of not only mitochondrial networks, but also other cellular organelles. We discovered that OXPHOS^HI^ LUAD cells contained a vast number of lipid droplets (LDs) that were nearly absent in OXPHOS^LO^ LUSC cells (Fig. 4a,b). Integrated throughout these LDs was a subpopulation of mitochondria in OXPHOS^HI^ LUAD cells that contacted single or clustered LDs to form peri-droplet mitochondria (PDM; Fig. 3a bottom left panel). PDM have been identified in brown adipose tissue^22^, heart^23^ and skeletal muscle^24^ but have not been described in lung cancer. In brown adipose tissue, PDM were characterized by elevated rates of mitochondrial respiration (that is, OXPHOS) versus cytoplasmic mitochondria (CM)^25^.

**Extended Data Fig. 10:**
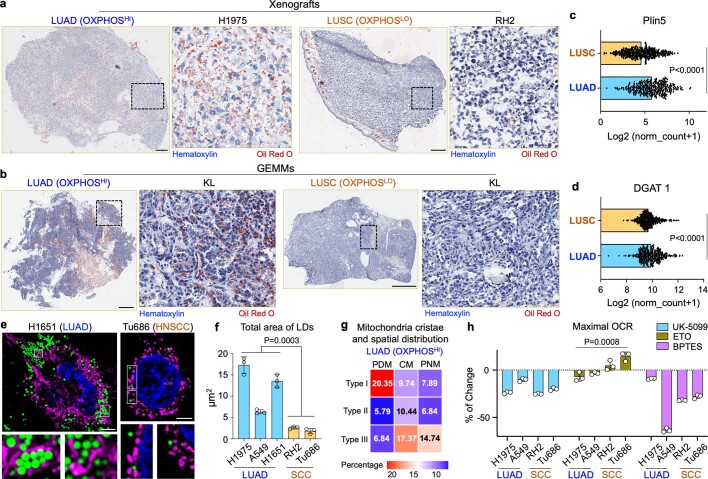
Differential accumulation of lipid droplets (LDs) between OXPHOS^HI^ LUAD and OXPHOS^LO^ SCC. **a,b**, Co-staining of oil red o and hematoxylin in OXPHOS^HI^ LUAD and OXPHOS^LO^ LUSC tumors from xenografts of human cells (**a**: H1975, RH2) and GEMMs (**b**: KL). Scale bar = 400 μm. **c,d**, The expression levels of Plin5 and DGAT1 in LUAD and LUSC tumors from The Cancer Genome Atlas (TCGA) analysis (n = 364 LUAD cells, n = 527 LUSC cells). Unpaired two-tailed t-test. **e**, Representative confocal Airyscan images stained with MitoTracker DeepRed (MTDR, purple), bodipy (green) and Hoechst (blue) in cultured human LUAD (H1651) and HNSCC (Tu686) cells. Scale bar = 5 μm. **f**, Quantification of total area of LDs per cell in human LUAD and SCC cells. Data are mean ± s.e.m. (n = 3 biological replicates, n > 300 cells per cell line). Unpaired two-tailed t-test. **g**, Heat map showing the percentage of mitochondrial population classified by both spatial distribution and cristae types in OXPHOS^HI^ LUAD cells. Data are mean value from n = 3 biological replicates (n > 1,200 mitochondria). **h**, Percentage of change in maximal OCR of human LUAD and SCC cells in response to UK-5099, ETO and BPTES. Data are mean ± s.e.m. (n = 3 biological replicates), unpaired two-tailed t-test. Source data

On the basis of the presence of PDM in OXPHOS^HI^ LUAD cells, we asked whether mitochondria may be organized into discrete subpopulations within these tumours. Using SBEM images from mouse LUAD and LUSC tumours, we grouped mitochondria into subpopulations based on their interaction with cellular organelles. These subpopulations include: PNM, localized to the nucleus; PDM that contacted LDs; and CM that were scattered throughout the cytoplasm and lacked both nuclear and LD contacts. OXPHOS^HI^ LUAD cells had a similar distribution of PNM, PDM and CM populations compared to OXPHOS^LO^ LUSC cells, which were nearly absent of LDs (Fig. 4a,c). To confirm our observations in the SBEM images, we measured LDs in vivo in both NSCLC xenografts and GEMMs by oil red O staining of tumours to label neutral lipids (triglycerides and diacylglycerols). We found a significant enrichment in lipids in OXPHOS^HI^ LUAD cells that was absent in OXPHOS^LO^ SCC cells (Fig. 4d–f and Extended Data Fig. 10a,b).

We next investigated whether PDM were present in human NSCLC tumour subtypes. Analysis of The Cancer Genome Atlases for human LUAD and LUSC identified that gene expression of DGAT1, a regulator of LD biogenesis, and that of PLIN5, which regulates LD formation^26,27^, were significantly upregulated in LUAD versus LUSC (Extended Data Fig. 10c,d). We then stained the OXPHOS^HI^ LUAD cell lines H1975, H1651 and A549 and the OXPHOS^LO^ SCC cell lines RH2 and Tu686 with BODIPY to identify LDs. Our results showed that the OXPHOS^HI^ LUAD cell lines had a significant increase in LDs and PDM formation compared with OXPHOS^LO^ squamous cell lines (Fig. 4g–l and Extended Data Fig. 10e,f). These results demonstrate that human and mouse OXPHOS^HI^ LUAD cells had a significantly higher number of LDs and PDM compared with OXPHOS^LO^ squamous tumours.

Given the nanometre resolution of our SBEM images, we next examined the crista architecture in PDM from OXPHOS^HI^ LUAD cells. Representative 2D and 3D images from tumour volumes showed elongated mitochondria that were interlaced throughout LD clusters and contacted multiple LDs (Fig. 4j). Notably, PDM exhibited densely packed orthodox type I cristae that aligned vertically at the mitochondria–lipid contact sites (Fig. 4k and Extended Data Fig. 10g). The enrichment of PDM for more OXPHOS-proficient type I cristae agrees with the data in Fig. 3, as well as previous studies in brown adipose tissue^25^.

We next examined both OXPHOS activity and nutrient preferences for glucose, glutamine or fatty acids in PDM-rich versus PDM-deficient human OXPHOS^HI^ LUAD and OXPHOS^LO^ SCC cell lines. We measured the percentage of change in basal and maximal OCR following treatment with inhibitors of pyruvate metabolism (UK5099), glutamine metabolism (BPTES) or fatty acid oxidation (etomoxir) in the H1975 and A549 versus RH2 and Tu686 cell lines. Our results showed that PDM-enriched LUAD cell lines utilize pyruvate, glutamine and fatty acid oxidation to support respiration, whereas PDM-deficient squamous cell lines were reliant on pyruvate and glutamine inhibition, but not fatty acid oxidation (Fig. 4l and Extended Data Fig. 10h).

Subsequently, we sought to understand whether PDM-rich NSCLC cells relied on different nutrient sources compared to PDM-deficient ones. We examined whether restriction of nutrients (glucose, glutamine or free fatty acids would limit growth of H1975 or RH2 cells. Our results showed that restricting free fatty acids significantly inhibited growth of H1975 cells but not RH2 cells (Fig. 4m). Both cell types were highly dependent on glutamine, as previously described in lung tumour cells^28^. H1975 cells grew well in low-glucose conditions, whereas RH2 cells did not grow, agreeing with previous studies that identified LUSC are reliant on both glucose and glutamine for cell survival^11,29^. In sum, we discovered PDM as a mitochondrial subpopulation that were enriched in OXPHOS^HI^ LUAD cells. Our data suggest that PDM-rich LUAD cells relied on a broad source of nutrients to support OXPHOS and growth whereas nutrient dependency in PDM-deficient squamous tumour cells was restricted to glucose and glutamine metabolism.

## Glucose regulation of mitochondrial motility

**Fig. 5:**
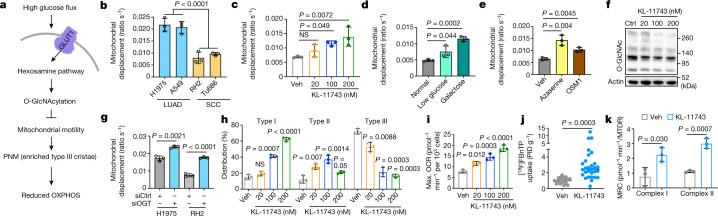
Glucose flux regulates mitochondrial motility and remodels crista structure through hexosamine pathway in OXPHOS^LO^ LUSC. Data are mean ± s.e.m. (*n* = 3 biological replicates), unpaired two-tailed *t*-test unless specified otherwise. **a**, Diagram of proposed model that glucose flux regulates the remodelling of mitochondrial cristae and reduction of OXPHOS function through hexosamine pathway. **b**–**e**, Basal mitochondrial displacement in human LUAD (H1975 and A549) and SCC (RH2 and Tu686) cells (**b**) and vehicle (Veh)- or treatment-driven mitochondrial displacement in RH2 cells (**c**–**e**). RH2 cells were treated with KL-11743 at indicated concentrations for 72 h (**c**), low-glucose (5.5 mM) and galactose medium for 24 h (**d**), or the hexosamine pathway inhibitors azaserine (0.5 μM) and OSM1 (25 μM) for 72 h (**e**). *n* = 150 per cell line or per treatment condition. One-way ANOVA, Dunnett test (**c**). **f**, Western blots of RH2 cells treated with indicated concentrations of KL-11743 for 72 h probed with indicated antibodies. **g**, Mitochondrial displacement in RH2 and H1975 cells treated with Ctrl siRNA (siCtrl) and *OGT* siRNA (siOGT) for 72 h. *n* = 150 per treatment condition. **h**, Percentage of type I, II and III cristae in RH2 cells treated with indicated concentrations of KL-11743. *n* > 1,500 mitochondria. One-way ANOVA, Dunnett test. **i**, Mitochondrial maximal OCR in RH2 cells treated with indicated concentrations of KL-11743 for 72 h. One-way ANOVA, Dunnett test. **j**,**k**, [^18^F]FBnTP uptake (**j**) and complex I and II MRC (**k**) of subcutaneous xenografts of human LUSC (RH2) cells treated with vehicle or KL-11743 (100 mg kg^−1^, 10 days). (**j**) *n* = 22 tumours for Veh; *n* = 29 tumours for KL-11743. Source data

**Extended Data Fig. 11:**
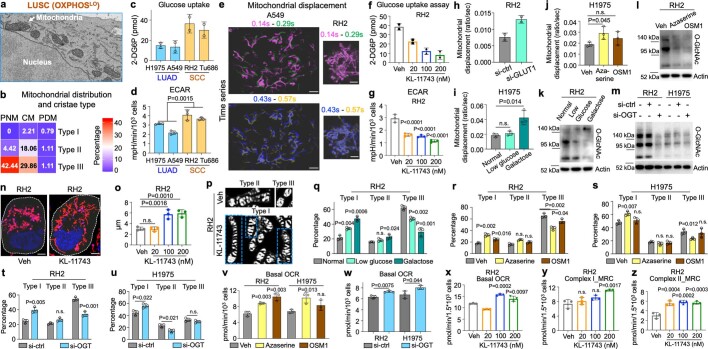
Glycolytic LUSC cells rescue OXPHOS activity by glucose restriction and inhibition of hexosamine pathway. Data are mean ± s.e.m. (n = 3 biological replicates), unpaired two-tailed t-test unless specified. **a**, Representative 2D SBEM image showing PNM and associated type III cristae in OXPHOS^LO^ and glycolytic LUSC. **b**, Heat map showing the percentage of mitochondrial population classified by both spatial distribution and cristae types in OXPHOS^LO^ LUSC cells. Data are mean value from n = 3 biological replicates (n > 750 mitochondria). **c,d**, Colorimetric assay measuring glucose uptake (**c**) and ECAR rate (**d**) in human LUAD (H1975, A549) and SCC (RH2, Tu686) cells. (**c**) n = 2 biological replicates. **e**, Representative of mitochondrial displacement by overlaying images from different time points in OXPHOS^HI^ LUAD (A549) and OXPHOS^LO^ LUSC (RH2) cells. Scale bar = 3 μm. **f,g**, Colorimetric assay measuring glucose uptake (**f**) and ECAR rate (**g**) in RH2 cells treated with vehicle (Veh) or KL-11743 with indicated concentrations for 72 h. (**f**) n = 2 biological replicates. (**g**) One-way ANOVA, Dunnett test. **h**, Mitochondrial displacement in RH2 cells treated with si-ctrl and si-GLUT1 for 72 h (n = 2 biological replicates, n > 60 per treating condition). **i,j**, Mitochondrial displacement in H1975 cells treated with low glucose (5.5 mM) and galactose medium for 24 h (**i**), hexosamine pathway inhibitors azaserine (0.5 μM) and OSM1 (25 μM) for 72 h (**j**). n > 100 per treating condition. **k–m**, Western blots were probed with indicated antibodies on lysates of RH2 cells treated with low glucose (5.5 mM) and galactose medium for 24 h (**k**), hexosamine pathway inhibitors azaserine (0.5 μM) and OSM1 (25 μM) for 72 h (**l**), and on lysates of RH2 and H1975 cells treated with si-ctrl and si-OGT for 72 h (**m**). **n**, Representative of confocal Airyscan imaged RH2 cells stained with MTDR (red) and Hoechst (blue) of after treatment of vehicle (Veh) or KL-11743 (200 nM) for 72 h, scale bar = 4 μm. **o**, Quantification of the spatial distribution of mitochondrial network in RH2 cells treated with Veh or KL-11743 with indicated concentrations. n = 300 cells per treating condition. One-way ANOVA, Dunnett test. **p**, Representative of confocal Airyscan imaged cristae structure (type I, II and III) in RH2 cells treated with Veh or KL-11743 (200 nM, 72 h) and stained with 10-N-nonyl acridine orange (NAO) and followed by Weka segmentation (ImageJ). **q–u**, Percentage of type I, II, III cristae in RH2 and H1975 cells treated with low glucose (5.5 mM) and galactose medium for 24 h (**q**), hexosamine pathway inhibitors azaserine (0.5 μM) and OSM1 (25 μM) for 72 h (**r,s**) and si-ctrl and si-OGT for 72 h (**t,u**). n > 600 mitochondria per treating condition. **v,w**, Mitochondrial basal OCR in RH2 and H1975 cells treated with hexosamine pathway inhibitors azaserine (0.5 μM) and OSM1 (25 μM) for 72 h (**v**) and si-ctrl and si-OGT for 72 h (**w**). **x–z**, Mitochondrial basal OCR (**x**) and Complex I (**y**) and Complex II (**z**) MRC in RH2 cells treated with indicated concentrations of KL-11743 for 72 h. One-way ANOVA, Dunnett test. Source data

Mitochondria move along the cytoskeleton (microtubules, actin and intermediate filaments) aided by motor, adaptor and transmembrane proteins^30,31^. Notably, motor adaptors sense glucose flux through O-GlcNAcylation by *O*-GlcNAc transferase (OGT). Here glucose is shunted into the nutrient-sensing hexosamine biosynthetic pathway, which activates OGT resulting in arrest of mitochondrial motility^32^. High glucose flux is a hallmark of hypermetabolic squamous tumours of the lung, head and neck as evidenced by elevated [^18^F]FDG uptake^11,29^ (Fig. 1e). We asked whether the perinuclear localization of mitochondria identified in hypermetabolic SCC cells was due to suppression of mitochondrial trafficking along the cytoskeleton as a result of high glucose flux (Fig. 3b and Extended Data Fig. 11a,b). We thus proposed a model in hypermetabolic SCC cells in which high glucose flux serves to confine mitochondria to the nucleus by repression of mitochondrial motility through activation of the hexosamine and OGT pathways (Fig. 5a).

We measured mitochondrial motility at basal levels or following inhibition of glucose flux in hypermetabolic, glycolytic RH2 and Tu686 versus less glycolytic H1975 and A549 cell lines^11^ (Fig. 1e). Mitochondrial motility was quantified by measuring the displacement of individual mitochondria over time, as previously described^33^. Basal glucose uptake and extracellular acidification rate was notably increased, whereas mitochondrial motility was significantly decreased in RH2 and Tu686 versus H1975 and A549 cell lines (Fig. 5b, Extended Data Fig. 11c–e and Supplementary Videos 9 and 10). We next measured mitochondrial motility following inhibition of glucose flux or the hexosamine pathway. Glucose uptake was restricted by modification of cell culture medium (low glucose (12 mM) or galactose), targeted inhibition of glucose transport through the pan GLUT1 and GLUT3 inhibitor KL-11743 (ref. ^34^) or RNA-mediated interference knockdown of the GLUT1 transporter that all led to a marked increase in mitochondrial motility (Extended Data Fig. 11f–h). The hexosamine pathway was inhibited using azaserine, which inhibits glutamine-fructose-6-phosphate transaminase [isomerizing] 2 (GFPT2), and the OGT inhibitor OSMI-1, as previously shown^35^. Inhibition of glucose uptake and the hexosamine pathway induced a significant increase in mitochondrial motility in RH2 and to a lesser extent H1975 cells (Fig. 5c–e and Extended Data Fig. 11i,j).

Next we investigated the role of OGT in the regulation of mitochondrial motility in NSCLC cells. In RH2 cells, glucose restriction, via treatment with KL-11743, azaserine, OSMI-1 or RNAi-mediated *OGT* knockdown, led to a decrease in total cellular O-GlcNAcylation (Fig. 5f and Extended Data Fig. 11k–m). Additionally, *OGT* knockdown significantly increased mitochondrial motility in RH2 and H1975 cells (Fig. 5g). In sum, these results demonstrate that glucose flux regulated mitochondrial motility in NSCLC tumour cells through the hexosamine pathway and OGT.

We then explored whether the increased mitochondrial motility as a result of glucose flux inhibition induced remodelling of mitochondrial networks. In RH2 cells, KL-11743 treatment induced a redistribution of mitochondria from nuclear to cytoplasmic localization (Extended Data Fig. 11b,n,o). We discovered that inhibition of glucose flux induced remodelling of crista architecture marked by a significant increase in type I cristae and a concomitant decrease in type III cristae in RH2 cells (Fig. 5h and Extended Data Fig. 11p,q). Glucose restriction, inhibition of the hexosamine pathway and *OGT* knockdown in RH2 and/or H1975 cells yielded similar results to KL-11743 treatment (Extended Data Fig. 11r–u).

The enrichment of well-formed orthodox type I cristae in NSCLC cells following crista remodelling suggested that OXPHOS activity may be upregulated following inhibition of glucose flux and/or the hexosamine–OGT pathway. We measured the maximal OCR in tumour cells following inhibition of glucose flux, the hexosamine pathway and OGT and these all induced a significant increase in basal and maximum OCR, as well as the complex I and II MRC in RH2, and to a lesser extent in H1975 cells (Fig. 5i and Extended Data Fig. 11v–z). Last, we examined OXPHOS signatures in vivo by carrying out [^18^F]FBnTP PET imaging on RH2 tumour xenografts treated with KL-11743 or vehicle. KL-11743 induced a significant increase in [^18^F]FBnTP uptake in RH2 tumours versus vehicle (Fig. 5j). Ex vivo respirometry carried out on tumours showed that KL-11743 treatment induced a significant increase in complex I and II MRC versus vehicle (Fig. 5k). These results demonstrate that inhibition of glucose flux and the hexosamine–OGT pathway led to significantly increased mitochondrial motility, enrichment of type I cristae and the upregulation of OXPHOS activity in glycolytic squamous tumours and to a lesser extend in LUAD cells.

**Extended Data Fig. 12:**
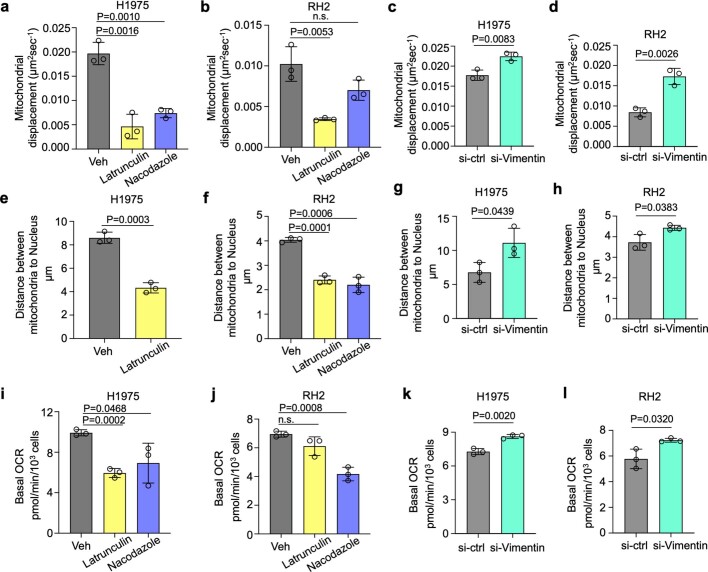
Mitochondrial motility, spatial distribution and respiration in OXPHOS^HI^ LUAD and OXPHOS^LO^ LUSC cells treated with the cytoskeleton disruptors. Data are mean ± s.e.m. (n = 3 biological replicates), unpaired two-tailed t-test unless specified. **a-d**, Mitochondrial displacement in H1975 (**a,c**) and RH2 (**b,d**) cells treated with latrunculin A (1 μM) and nocodazole (6.7 μM) for 12 h (**a,b**), and treated with si-ctrl and si-Vimentin for 72 h (**c,d**). n > 90 cells per treating condition. **e-h**, Quantification of the spatial distribution of mitochondrial network in H1975 (**e,g**) and RH2 (**f,h**) cells treated with latrunculin A (1 μM) and nocodazole (6.7 μM) for 12 h (**e,f**), and treated with si-ctrl and si-Vimentin for 72 h (**g,h**). n > 150 cells per treating condition. **i–l**, Mitochondrial basal OCR in H1975 (**i,k**) and RH2 (**j,l**) cells treated with latrunculin A (1 μM) and nocodazole (6.7 μM) for 12 h (**i,j**), and treated with si-ctrl and si-Vimentin for 72 h (**k,l**). Source data

Last, we investigated the role of microtubules, actin and vimentin in the regulation of mitochondrial motility in H1975 and RH2 NSCLC cell lines. Disruption of both microtubule and actin networks by treatment with latrunculin A or nocodazole, respectively, led to a notable decrease in mitochondrial motility in both cell lines (Extended Data Fig. 12a,b). By contrast, siRNA-mediated knockdown of vimentin led to a significant increase in mitochondrial motility (Extended Data Fig. 12c,d). We next confirmed that both latrunculin A and nocodazole treatment induced a significant decrease in the distance of mitochondria from the nucleus resulting in an enrichment of PNM, whereas knockdown of vimentin led to a significant increase (Extended Data Fig. 12e–h). Last, we measured basal OCR after disruption of the components of the cytoskeleton and identified that latrunculin A and nocodazole treatment induced a notable decrease in basal OCR whereas vimentin knockdown induced a significant increase (Extended Data Fig. 12i–l). These results indicate that both microtubules and active networks support mitochondrial motility, cytoplasmic distribution of mitochondria and respiratory capacity whereas vimentin functions in an antagonistic and suppressive manner.

## Discussion

In summary, we present an in vivo structural and functional analysis of mitochondrial networks and bioenergetic phenotypes across mouse and human NSCLC tumours. The ex vivo respirometry we carried out on NSCLC tumours identified significant increases in complex I and complex II respiratory activity in [^18^F]FBnTP-positive LUAD cells, whereas glycolytic LUSC cells with low [18F]FBnTP uptake had persistently low complex I activity. Supporting this, it was recently shown that ubiquinol oxidization is required for lung tumour growth, emphasizing a critical role of complex III and the ETC in lung tumorigenesis^36^. These results underscore the diversity of bioenergetic activity among NSCLC tumour subtypes. The functional diversity we discovered between LUAD cells and LUSC cells accurately predicted distinct structural mitochondrial phenotypes among lung tumour subtypes. PET-guided 3D SBEM, combined with CNN machine learning analysis, facilitated the large-scale mapping of the structural and spatial distribution of mitochondrial networks within [^18^F]FBnTP-positive LUAD cells and [^18^F]FBnTP-negative LUSC cells. The broad diversity of mitochondrial structures identified in LUAD and LUSC tumours correlated with equally diverse bioenergetic profiles and metabolic dependencies in these histological subtypes. In addition, we identified a previously unrecognized compartmentalization of mitochondrial subpopulation in LUAD cells in which PDM populations were enriched in these tumours.

The closely regulated and uniform organization of mitochondrial networks we profiled in LUSC cells corresponded with reduced metabolic flexibility compared with LUAD cells. LUSC were more reliant on glucose and glutamine metabolism to support OXPHOS and less so on fatty acid oxidation, whereas LUAD cells utilized glucose, glutamine and fatty acids to support cellular respiration. These results agree with previous studies that demonstrated LUSC cells rely on glucose and glutamine to support tumour metabolism and are selectively sensitive to inhibition of these pathways^11,28^. Structure–function studies defining the relationship between mitochondrial architecture and metabolic dependencies may hold promise as an emerging diagnostic and therapeutic strategy that can be leveraged to exploit bioenergetic and metabolic liabilities unique to lung cancer subtypes. We anticipate that coupling PET imaging with 3D SBEM will have dynamic applications beyond that of lung cancer and enrich our understanding of how mitochondrial bioenergetics impact human disease.

## Methods

### Cell culture

Cells were cultured in Dulbecco’s modified Eagle’s medium (Thermo Fisher Scientific) supplemented with 10% fetal bovine serum (Hyclone) and 1% penicillin/streptomycin (Gibco). A549 cells and H1975 cells were obtained from ATCC. The lung squamous cell line (human) RH2 were established in the laboratory of S.M.D. (UCLA). The head and neck squamous cell line (human) Tu686 was a gift from the laboratory of M.S.J. (UCLA). The mouse lung squamous cell line derived from mouse 5 (LPP) and mouse LUAD cell line derived from mouse 4 (LPP) were established in our laboratory. After resecting the tumour tissues, they were minced with razors and digested with collagenase/dispase (Sigma). Lysate was filtered through a 70-μm cell strainer. Dissociated single cells were centrifuged and resuspended in Dulbecco’s modified Eagle’s medium (10% fetal bovine serum). The cells were plated in tissue culture dishes and the medium was changed until there were enough colonies to expand the culture. All cells were grown at 37 °C in 5% CO_2_ in a humidified incubator, and a test for mycoplasma was carried out using the LookOut Mycoplasma PCR Detection Kit (Sigma). Cell identities were confirmed by Laragen Inc. The endoribonuclease-prepared siRNA used in this study was: *GLUT1* (*SLC2A1*) (EHU028011, Sigma); *OGT* (EHU082301, Sigma).

### GEMMs of lung tumour

We used five GEMMs in this study: (1) Kras-Lox-Stop-Lox-G12D; Rosa26-Lox-Stop-Lox-Luc (Kras); (2) Kras-Lox-Stop-Lox-G12D; LKB1 Lox/Lox; Rosa26-Lox-Stop-Lox-Luc mice (KL); (3) Kras-Lox-Stop-Lox-G12D; P53 Lox/Lox; Rosa26-Lox-Stop-Lox-Luc mice (KP); (4) Kras-Lox-Stop-Lox-G12D; LKB1 Lox/Lox; P53 Lox/Lox; Rosa26-Lox-Stop-Lox-Luc (KPL); (5) LKB1 Lox/Lox; P53 Lox/Lox; PTEN Lox/Lox; Rosa26-Lox-Stop-Lox-Luc (LPP). Lung tumours were induced by Ad5-CMV-Cre (VVC-U of Iowa-1174) or LentiCre (Kerafast) delivered intranasally as described previously^37^. Tumour growth was routinely monitored by bioluminescence imaging using an IVIS imager (PerkinElmer). All animal experiments were approved by UCLA’s Animal Research Committee (ARC) and carried out following ARC protocols and requirements. The tumour burden endpoints (morbidity; weight loss no greater than 20%; laboured breathing; impingement of animal’s ability to ambulate, eat and drink) allowed by our Institutional Animal Care and Use Committee were not exceeded. Lung tumours from different GEMM mice were collected and snap frozen in liquid nitrogen. Snap-frozen samples were stored at −80 °C until respirometry assay and western blotting analysis.

### Subcutaneous implantation in NSG mice

A549, H1975, A549 Rho, RH2 and Tu686 cells were cultured in vitro under the conditions described above. Cells were collected and suspended in PBS, then mixed with Matrigel Membrane Matrix (Corning) and implanted subcutaneously on the flanks of NSG mice (2–4 × 10^6^ cells per flank). All animal experiments were approved by UCLA’s ARC and carried out following ARC protocols and requirements. The tumour burden endpoints (tumour volume no greater than 2,000 mm^3^; morbidity; weight loss no greater than 20%; laboured breathing; impingement of animal’s ability to ambulate, eat and drink) allowed by our Institutional Animal Care and Use Committee were not exceeded. For the treatment study of the GLUT1 and GLUT3 inhibitor KL-11743 (C6), mice were treated with C6 (100 mg kg^−1^) delivered by oral gavage for 8 days. Subcutaneous tumours were either snap frozen in liquid nitrogen or fixed in 10% formalin overnight. Snap-frozen samples were stored at −80 °C until respirometry assay. Formalin-fixed samples were sent to the Translational Pathology Core Laboratory at UCLA for embedding and sectioning.

### [^18^F]FBnTP synthesis

Synthesis of the radioactive [^18^F]FBnTP probe was carried out as previously described^9,38^.

### PET–CT imaging

PET–CT imaging and analysis were carried out on GNEX as previously described^37,39^. PET signals were measured as percentage of injected dose per gram after 1 h uptake and normalized to heart signal. Tumours with a tumour-to-heart [^18^F]FBnTP uptake ratio of ≥0.5 are defined as [^18^F]FBnTP^HI^; those with a ratio of <0.5 are defined as [^18^F]FBnTP^LO^. Tumours with a tumour-to-heart [^18^F]FDG uptake ratio of ≥0.2 are defined as [^18^F]FDG^HI^; those with a ratio of <0.2 are defined as [^18^F]FDG^LO^ (ref. ^26^).

### Ex vivo respirometry analysis on frozen tissues

Tumour tissues were isolated from GEMMs of lung cancer and snap frozen using liquid nitrogen. Frozen samples were stored in −80 °C until use in the Seahorse experiments. Frozen tissues were thawed on ice and homogenized in MAS buffer (70 mM sucrose, 220 mM mannitol, 5 mM KH_2_PO_4_, 5 mM MgCl_2_, 1 mM EGTA, 2 mM HEPES pH 7.4) with protease inhibitor cocktail (Roche). Homogenates were centrifuged at 1,000*g* for 10 min at 4 °C and supernatant was collected. Protein concentrations were determined by BCA assay kit (Thermo Fisher). Homogenates (12 µg per well for human samples and 6 µg per well for mouse samples) were loaded into a Seahorse XF96 microplate in MAS buffer (20 µl each well) and centrifuged at 2,000*g* for 5 min at 4 °C. After centrifugation, the volume was increased to 150 µl by adding 130 µl MAS containing cytochrome *c* (10 µg ml−1). At port A, substrates of NADH (1 mM) were injected to determine the respiratory capacity of mitochondrial complex I; succinate (5 mM) + rotenone (2 µM) were injected to determine the respiratory capacity of mitochondrial complex II. The following compounds were injected so that final concentrations were as follows—port B: rotenone (2 µM) + antimycin (4 µM); port C: TMPD (0.5 mM) + ascorbic acid (1 mM); port D: azide (50 mM). OCR rates were measured using a Seahorse XF96 Extracellular Flux Analyzer (Agilent Technologies) and normalized to mitochondrial content quantified by MTDR. Homogenates were stained with 500 nM MTDR for 10 min followed by two wash steps to remove the dye (Thermo Fisher). MTDR fluorescence was read on a Tecan Spark plate reader (Ex: 633 nm; Em: 678 nm).

### In vitro respirometry analysis on cultured cells

Cells were seeded into a Seahorse XF96 microplate before the assay and maintained in a tissue culture incubator (37 °C in 5% CO_2_) to reach 90–100% density before running the Seahorse assay. To measure complex I and complex II respiratory capacity, cells were permeabilized with 4 nM XF plasma membrane permeabilizer (Agilent). Permeabilized cells were started in state 3 with substrates of pyruvate (5 mM) + malate (0.5 mM) and 4 mM ADP for respiration-driven through complex I; succinate (5 mM) + rotenone (2 µM) and 4 mM ADP for complex II. Following state 3 measurements, injections included the following—port A: oligomycin (2 µM); ports B and C: FCCP (B: 0.75 µM and C: 1.35 µM); port D: rotenone (1 µM) + antimycin (2 µM). To measure OCR and extracellular acidification rate in intact cells, cells were washed twice and incubated with freshly prepared assay medium (Seahorse XF Base Medium + 2 mM l-glutamine + 1 mM pyruvate + 10 mM glucose) for 30 min. The following compounds were injected in the order of oligomycin (2 µM), FCCP (0.75/1.35 µM), rotenone (1 µM) + antimycin A (2 µM). Cell count (per well) was determined by the number of nuclei stained with Hoechst (10 µg ml^−1^) and quantified by an Operetta High-Content Imaging System (PerkinElmer). OCR and extracellular acidification rate were normalized to cell count per well. For nutrient-dependent respirometry analysis, conditions of UK5099 (5 µM), BPTES (3 µM) and etomoxir (3 µM) were applied.

### Whole-animal perfusion and tissue fixation

Fixative solution (2% paraformaldehyde, 2.5% glutaradehyde, 0.15 M cacodylate and 2 mM Ca^2+^) was freshly prepared every time. Animals were anaesthetized by ketamine (200 mg kg^−1^) and xylazine (10 mg kg^−1^) delivered by intraperitoneal injection. Anaesthesia took effect after several minutes. The depth of anaesthesia was tested using tail pinch and paw prick, and it was ensured that the breathing did not stop. The body cavity was cut with scissors, and cut up the midline to the sternum. The heart was exposed and a needle was placed into the left ventricle and then the right atrium was snipped with iridectomy scissors. The animal was perfused for 30 s at the flow speed of 8 ml min^−1^ with Ringer’s solution supplemented with 2% xylocaine and 1,000 U herapin. Then the valve on the pump was switched on to fixative solution and perfusion was carried out for 5–8 min. After continuous perfusion, mouse lung tumour was collected, placed in ice-cold fixative solution and fixed overnight in the fridge. Fixed tissues were washed three times with 0.15 M cacodylate buffer (2 mM Ca^2+^) and stored in the same buffer at 4 °C until further processing.

### Sample preparation for SBEM imaging

Post-fixed tissues of mouse lung tumour were washed in 0.15 M cacodylate buffer (2 mM Ca^2+^). Tissues were stained for 1 h with 2% osmium and 1.5% potassium ferrocyanide in 0.15 M cacodylate buffer (2 mM Ca^2+^). Tissues were washed five times (5 min each time) with double-distilled (dd)H_2_O, and then placed in filtered TCH buffer (0.05 g thiocarbohydrazide in 10 ml ddH_2_O) for 20 min at room temperature. Tissues were washed five times (5 min each time) with ddH_2_O and then stained with 2% osmium in ddH_2_O for 30 min at room temperature. Next, tissues were stained with 2% uranyl acetate overnight at room temperature and lead aspartate solution (0.66% (w/v) lead in 0.03 M aspartic acid) for 30 min in a 60 °C oven. Tissues were dehydrated in serial ice-cold ethanol (70%, 90% and 100%) and ice-cold acetone after washing in ddH_2_O. Tissues were then embedded in serial Durcupan resin (50%, 75%, 100%) and solidified in a 60 °C oven for 2 days.

### SBEM

SBEM volumes were collected on a Zeiss Gemini 300 microscope equipped with a Gatan 3View 2XP microtome system. The volumes were collected at 2.5 kV using a 30-μm aperture, the gun in analytic mode, and the beam in high-current mode. Focal charge compensation with nitrogen gas was used to mitigate charging artefacts. The dwell time was 1 μs. The pixel size was either 5 or 6 nm, and the *Z* step size was always 50 nm. Following data collection, the images were converted to .mrc format and cross-correlation was used for rigid image alignment of the slices using the IMOD image processing package^40^.

### MicroCT

MicroCT imaging was carried out on a Zeiss Versa 510 microscope. The wet lung specimens were imaged with no contrast staining in buffer at 40 kV, using 360° of specimen rotation and 801 projection images were collected. The pixel size was 11.5 μm. Specimens stained for SBEM and embedded in epoxy were imaged at 80 kV, using 360° of specimen rotation and 1,601 projection images were collected. The pixel size was between 5 and 8 μm.

### 3D visualization and analysis of SBEM images

Individual cells were segmented using Amira software (Thermo Scientific). Sequential SBEM images were processed with median filtering and contrast adjustment to enhance the appearance of intracellular space. Interactive thresholding was applied to distinguish cellular and extracellular space and generate binary images. Binary images were processed with the separate object algorithm to fragment cells without clear extracellular boundaries. The connected components algorithm was applied to create individual labels for each identified cell. Segmented individual cells in each stack were visualized with a voxelization rendering.

### Machine-learning-based segmentation of nucleus and mitochondria in SBEM images

Our method segments nuclei (class 1) and mitochondria (class 2) from the background (class 0) in a 3D SBEM volume. The method takes individual slices from the volume (2D images) as input and predicts the segmentation for the slices independently. To carry out the segmentation, a deep CNN was used, which is a function that takes an image as input and outputs logits (or softmax confidence) corresponding to each class. The class with the highest response is chosen for each pixel to yield the segmentation map for each slice. Specifically, an encoder–decoder architecture based on U-Net^41^, with three modifications was chosen. First, ResNet^42^ blocks (with 32, 64, 128, 256 and 256 filters) was used instead of standard convolutional blocks. Second, as mitochondria are small objects, we chose to limit spatial downsampling to one-eighth of the original image. To do so, the max-pooling operation was removed after the third layer. Third, an atrous spatial pyramid pooling^43^ layer was used with strides 6, 12, 18 and 24 as the last layer of our encoder to increase the field of view of our filters. All weights use random uniform initialization, and we used leaky-ReLU activations. Our model is trained using the standard cross-entropy loss and is optimized using Adam^44^ with *β*_1_ = 0.9 and *β*_2_ = 0.999. We used an initial learning rate of 1 × 10^−4^ and decreased it to 1 × 10^−5^ after 150 epochs for a total of 200 epochs. We use a batch size of 8 and resize each image to 768 × 768. We carry out random horizontal and vertical flips, rotations between [−20°, 20°], zero-mean additive Gaussian noise with standard deviation of 0.08, and crop sizes up to 90% of the image height and width as data augmentation. Each augmentation was applied with a 50% probability. Our model was trained on ten densely labelled images, and was validated on three additional labeled images that are not included in the training set. Training takes about 8 h on an Nvidia GTX 1080 GPU, and inference takes about 11 ms per 2D image. Our segmentation model was evaluated using the Sorensen–Dice index (DICE), the Jaccard index (also referred to intersection-over-union, IoU), precision and recall. DICE and IoU are metrics to gauge the similarity of two sets, commonly used to evaluate segmentation results. Our segmentation model achieves an average of 0.92 DICE score, 0.86 IoU, 0.916 precision and 0.927 recall, as compared to that of manual registration.

### Mitochondrial motility and mitochondrial crista analysis

A total of 2 × 10^5^ cells were plated per well into CELLview 4-compartment glass-bottom tissue culture dishes (Greiner Bio-One) 48 h before the imaging section, and maintained in a tissue culture incubator (37 °C in 5% CO_2_). Cells were stained with TMRE (15 nM, Thermo Fisher) for 1 h to analyse mitochondrial motility, and with 10-*N*-nonyl acridine orange (100 nM, Thermo Fisher) for 1–3 h to analyse mitochondrial cristae. Live-cell imaging was carried out on the Zeiss LSM 880 with Airyscan using the alpha Plan-Apochromat 100×/1.46 Oil DIC M27 objectives. Images were deconvolved by Airyscan Processing in ZEN software. Image analysis was carried out using ImageJ (Fiji) in the order of: background subtraction, crop region of interest, adjust thresholds and measure parameters. Mitochondrial motility was analysed using a program developed in the laboratory of O.S.S. To measure mitochondrial motility, each field of view was imaged every 20 s, 10 times (yielding a total of 10 frames). Mitochondrial placement in frame 1 of each time series is identified as the reference point. The following 9 frames (*n* = 2, 3, 4…10) in the image set were then overlaid on the reference image, and the overlapping area between frame *n* and frame 1 was calculated and denoted as overlap_area(*n*). The non-overlapping mitochondria area between frame (*n*) and the reference image was denoted as travel_area(*n*) and calculated with the formula: travel_area(*n*) = mitochondria_area(*n*) − overlap_area(*n*). Mitochondrial motility was then calculated as the displacement ratio for each frame, ratio(*n*) = travel_area(*n*)/overlap_area(*n*). We then fitted a linear regression to the ratio for each image frame (*n* > 1) with the time intervals (20 s, 40 s…180 s), and the regression line is set in the form of ratio = *a* (slope) × time intervals + *b* (intercept). The value of “*a* (slope)” for each time series of image sets is our readout of mitochondrial motility. Macros were designed for automated segmentation and quantification of mitochondrial crista structure and density according to previously described methods^19,21^. The original images used for classification of mitochondrial cristae were 2D images, and the consensus for classification was obtained from two.

### Quantification of distance between mitochondria and nucleus in fluorescent images

Given the centre of a mitochondria, we want to find its minimum distance to the surface of a nucleus, which is segmented as an ellipse. The result can be obtained by solving a constrained optimization problem using Lagrange multipliers. The original ellipse of the nucleus is tilted by a degree and is centred at an arbitrary coordinate. To ease the computation, we translate and rotate the original ellipse to a standard ellipse that is centred at (0,0) with the major axis aligned with the *x* axis and the minor axis aligned with the *y* axis. The centre of the mitochondria is mapped to the new coordinate system accordingly. Through substitution, the gradient of the objective function that involves only *y* is a fourth-degree polynomial, which has at most four possible roots that satisfy the criteria. We then choose the coordinate (*x*,*y*) on the ellipse that has the minimal distance to the target nucleus.

### Immunohistochemistry

Immunohistochemistry staining and analysis were carried out as previously described^11^. Tissue slides were probed with the following antibodies: CD34 (1:800, ab8158 Abcam); TTF1 (1:1,000, M3575 Dako); CK5 (1:1,000, ab52635 Abcam).

### Oil red O staining

The oil red O staining was carried out following the protocols of a commercial kit (StatLab, KTOROPT). Frozen tissues were fixed by O.C.T compound (Fisher HealthCare) and sectioned at the Translational Pathology Core Laboratory (UCLA). The frozen tissue sections were fixed in 10% formalin for 5 min, and then rinsed in distilled water. Slides were immersed in propylene glycol for 2 min, followed by immersion in preheated oil red O solution at 60 °C for 6 min. Slides were then washed in 85% propylene glycol for 1 min and running distilled water for 1 min. The nuclei were stained with modified Mayer’s haematoxylin for 1 min and rinsed in distilled water for 1 min. The slides were mounted with aqueous medium and covered with a coverslip.

### Western blotting

Whole-cell lysates of lung tumours isolated from GEMMs were generated by homogenizing snap-frozen tumour tissues in lysis buffer (20 mM Tris pH 7.5, 150 mM NaCl, 1% (v/v) Triton X-100, 50 mM sodium fluoride, 1 mM EDTA, 1 mM EGTA, 2.5 mM pyrophosphate, 1 mM sodium orthovanadate, complete protease inhibitor cocktail). Whole-cell lysates were centrifuged at 2,000*g* for 5 min and supernatants were transferred to empty tubes. Supernatants were stored at −80 °C until use. Whole-cell lysates of in vitro-cultured cells were generated by homogenizing the cells SDS lysis buffer (100 mM Tris pH 7.5, 100 mM NaCl, 1% SDS, protease inhibitor cocktail) followed by heat inactivation at 90 °C for 10 min. Protein concentration was determined by BCA assay (Thermo Fisher). Lysates were run on 4–12% Bis-Tris gels (Thermo Fisher) to separate the proteins, and then transferred to PVDF membrane. Membranes were stained with Poceau S to confirm transfer efficiency. Membranes were then probed with the following antibodies: SP-C (1:5,000, AB3786 Millipore); GLUT1 (1:2,000, GT11-A, Alpha Diagnostic); NDUFS1 (1:1,000, ab169540, Abcam); *O*-linked *N*-acetylglucosamine (1:1,000, ab2739, Abcam); SDHA (1:1,000, 5839, Cell Signaling Technology); SDHC (1:1,000, ab155999, Abcam); actin (1:5,000, A3853, Sigma); tubulin (1:2,500, T9026, Sigma).

### Statistics and reproducibility

Statistical analyses were carried out on GraphPad Prism 9 or R studio. Differences between groups were determined using unpaired two-tailed *t*-test or one-way ANOVA if more than two groups were compared. For treatment studies, Dunnett’s test was used to compare every mean to a control mean. For non-treatment studies, Tukey’s test was used to compare every mean to every other mean. Data are presented as mean ± s.e.m. unless specified otherwise. Numbers of biological replicates are indicated in the figure legends. All experiments were repeated in at least duplicate. No statistical methods were used to predetermine sample size.

### Reporting summary

Further information on research design is available in the Nature Portfolio Reporting Summary linked to this article.

## Online content

Any methods, additional references, Nature Portfolio reporting summaries, source data, extended data, supplementary information, acknowledgements, peer review information; details of author contributions and competing interests; and statements of data and code availability are available at 10.1038/s41586-023-05793-3.

## Supplementary information

Supplementary Fig. 1 Source data for western blots.

Reporting Summary

Supplementary Video 1 3D-rendered microCT volume of lung lobe with tumour.

Supplementary Video 2 Tumour region selected for SBEM tumour volumes.

Supplementary Video 3 3D-rendered SBEM tumour volume.

Supplementary Video 4 3D-rendered partial SBEM tumour volume.

Supplemental Video 5 Slice-by-slice view of an SBEM tumour volume.

Supplementary Video 6 Colour-coded cellular segmentation in a 3D-rendered SBEM tumour volume.

Supplementary Video 7 Representative 3D images of PNM networks in LUSC cells.

Supplementary Video 8 Representative 3D images of mitochondrial networks in LUAD cells.

Supplementary Video 9 Mitochondrial motility within A549 cells.

Supplementary Video 10 Mitochondrial motility within RH2 cells.

## Data Availability

Source data for western blots are provided in Supplementary Fig. 1. Data that support the findings of this study have been deposited in the Cell Image Library (http://cellimagelibrary.org/groups/54862) or are available from the corresponding author upon reasonable request. Source data are provided with this paper.

## Source data

Source Data Fig. 1
Source Data Fig. 2
Source Data Fig. 3
Source Data Fig. 4
Source Data Fig. 5
Source Data Extended Data Fig. 1
Source Data Extended Data Fig. 2
Source Data Extended Data Fig. 4
Source Data Extended Data Fig. 5
Source Data Extended Data Fig. 7
Source Data Extended Data Fig. 8
Source Data Extended Data Fig. 9
Source Data Extended Data Fig. 10
Source Data Extended Data Fig. 11
Source Data Extended Data Fig. 12

## Code availability

The code for measuring mitochondrial parameters in SBEM image sets and confocal fluorescence image sets have been deposited in Github (https://github.com/tiard/mito-networks-3d).
