# Supplementary figures and images for: Spatial mapping of mitochondrial networks and bioenergetics in lung cancer
Source: Nature. 2023 Mar 15;615(7953):712–9. doi: 10.1038/s41586-023-05793-3 (PMC10033418; doi:10.1038/s41586-023-05793-3)

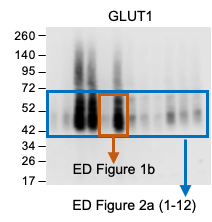

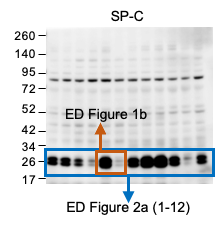


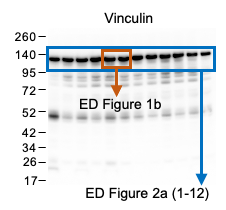


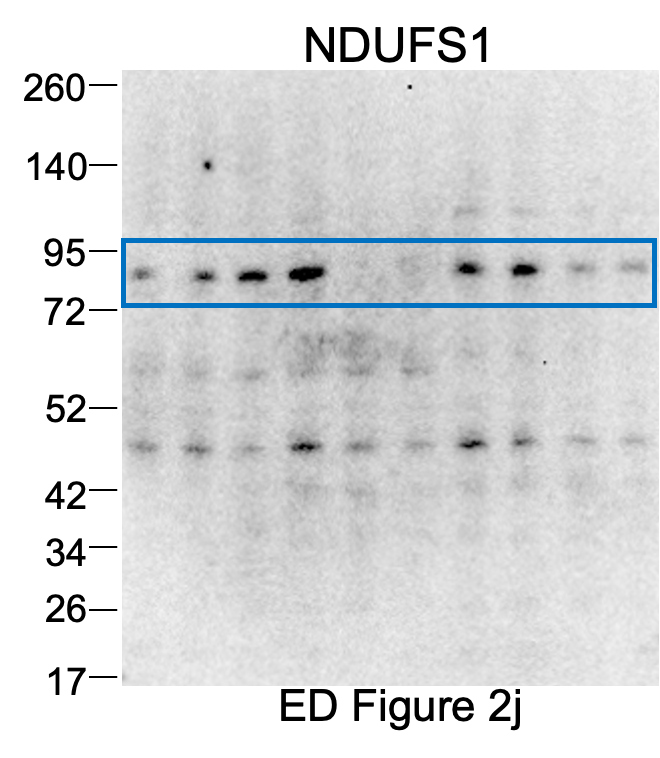


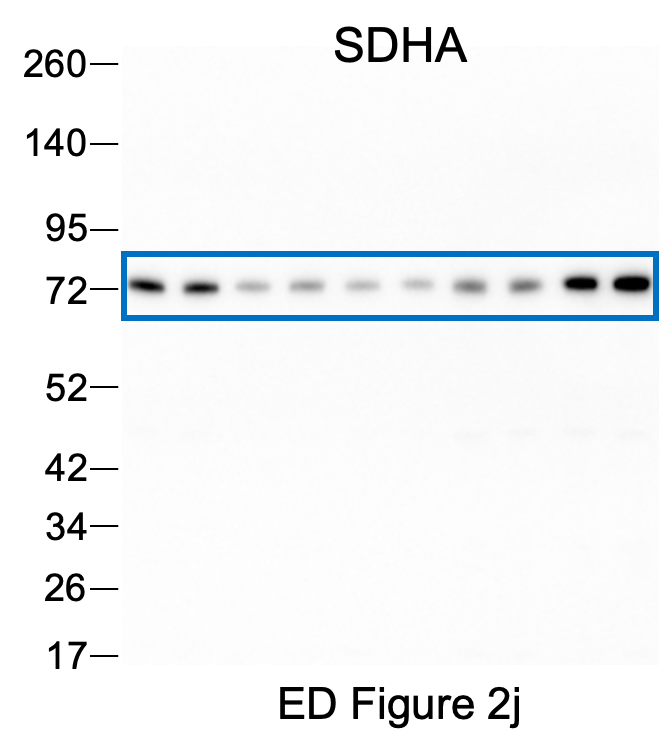

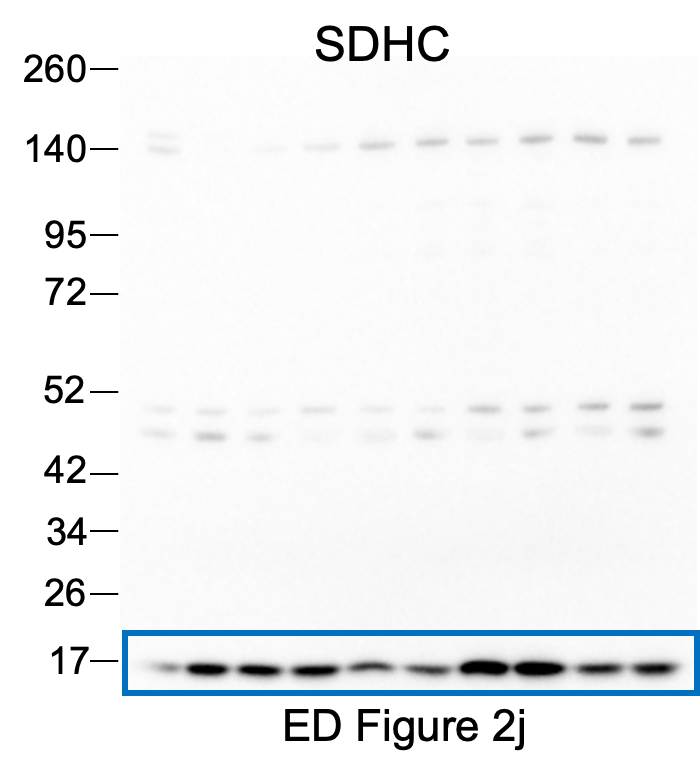


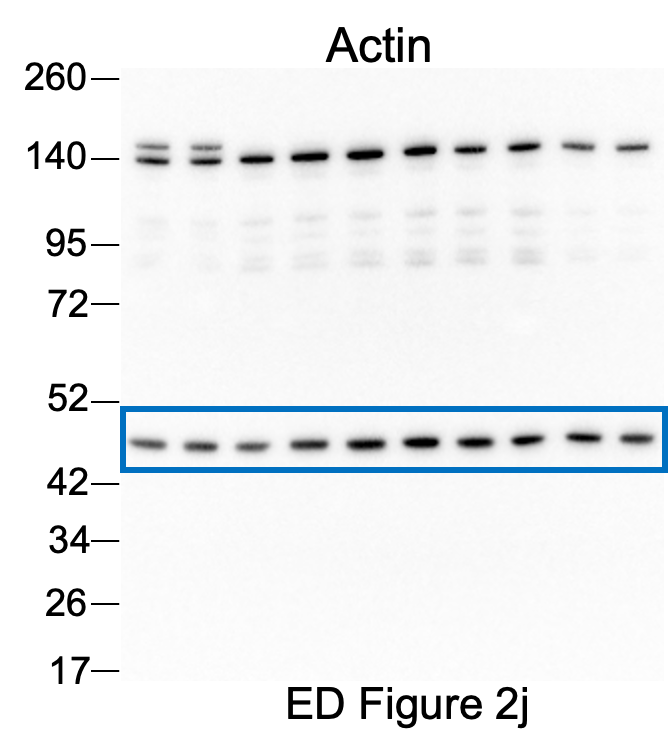

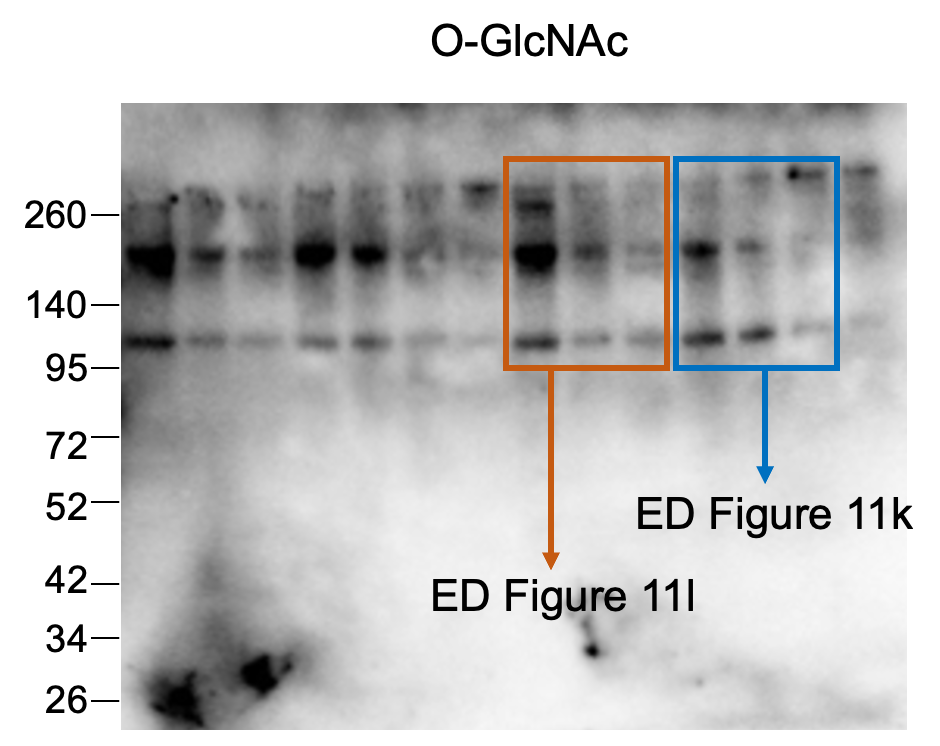


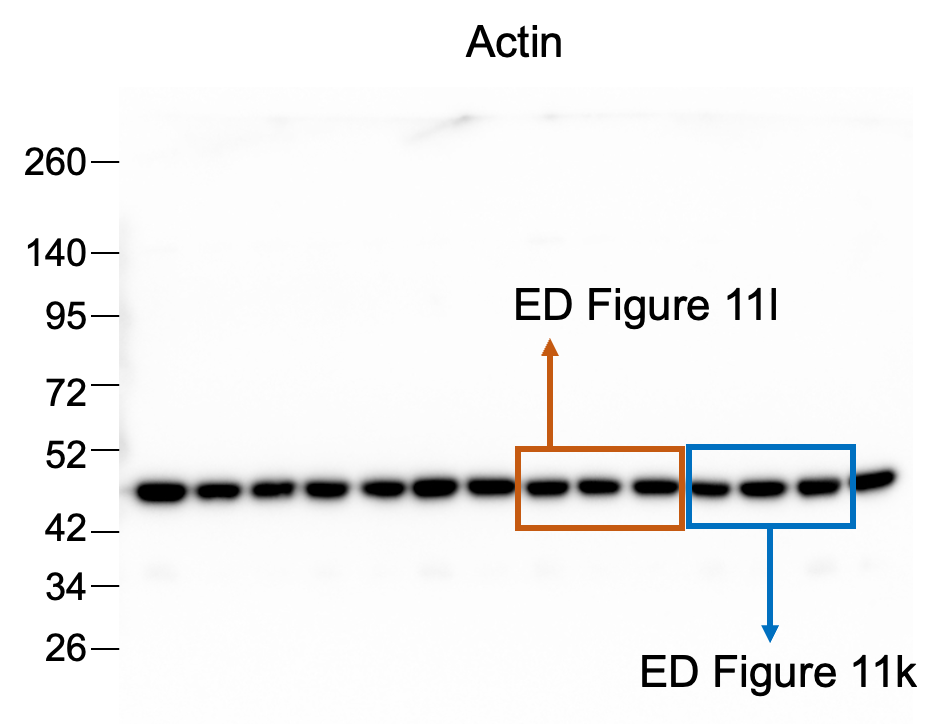

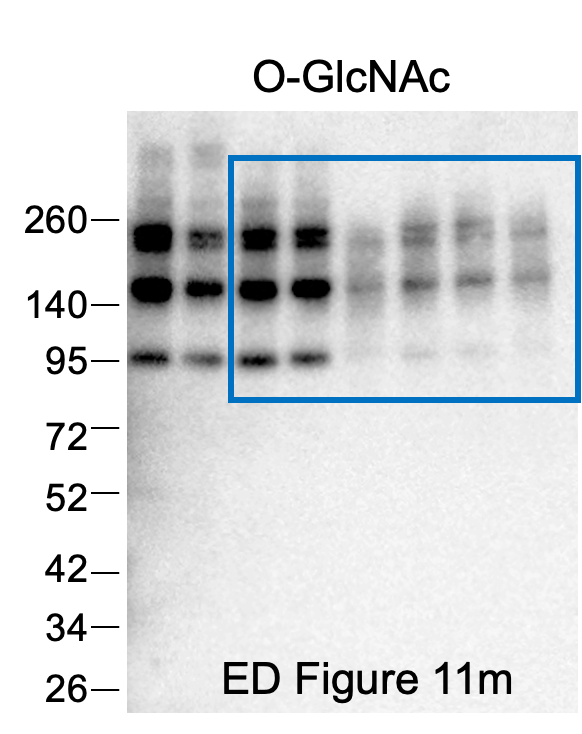


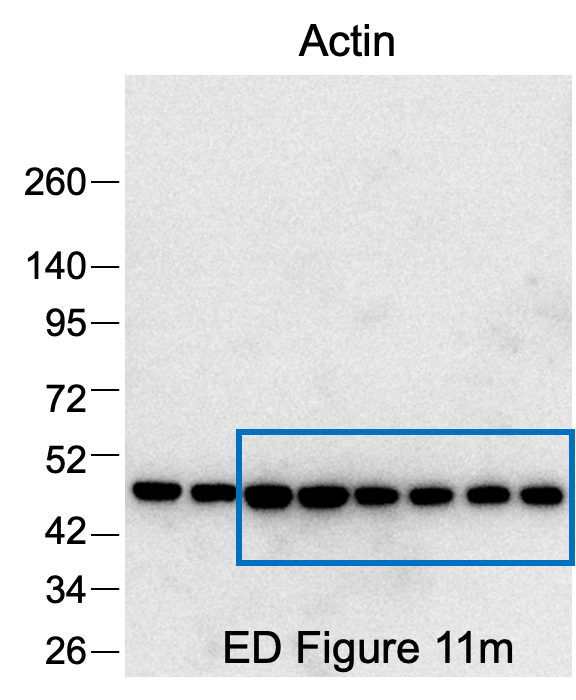

Supplement: Supplementary file 1 — Source data for western blots. [file 41586_2023_5793_MOESM1_ESM.docx]
