# Supplementary material for: Spatial mapping of mitochondrial networks and bioenergetics in lung cancer
Source: Nature. 2023 Mar 15;615(7953):712–9. doi: 10.1038/s41586-023-05793-3 (PMC10033418; doi:10.1038/s41586-023-05793-3)
Supplement: Supplementary file 2 — Reporting Summary [file 41586_2023_5793_MOESM2_ESM.pdf]

nature research

Corresponding author(s): David Shackelford

Last updated by author(s): 12/22/2022

Reporting Summary

Nature Research wishes to improve the reproducibility of the work that we publish. This form provides structure for consistency and transparency in reporting. For further information on Nature Research policies, see our Editorial Policies and the Editorial Policy Checklist.

Statistics

For all statistical analyses, confirm that the following items are present in the figure legend, table legend, main text, or Methods section.

n/a	Confirmed	
☐	☒	The exact sample size (*n*) for each experimental group/condition, given as a discrete number and unit of measurement
☐	☒	A statement on whether measurements were taken from distinct samples or whether the same sample was measured repeatedly
☐	☒	The statistical test(s) used AND whether they are one- or two-sided<br>*Only common tests should be described solely by name; describe more complex techniques in the Methods section.*
☒	☐	A description of all covariates tested
☐	☒	A description of any assumptions or corrections, such as tests of normality and adjustment for multiple comparisons
☐	☒	A full description of the statistical parameters including central tendency (e.g. means) or other basic estimates (e.g. regression coefficient) AND variation (e.g. standard deviation) or associated estimates of uncertainty (e.g. confidence intervals)
☐	☒	For null hypothesis testing, the test statistic (e.g. *F*, *t*, *r*) with confidence intervals, effect sizes, degrees of freedom and *P* value noted<br>*Give P values as exact values whenever suitable.*
☒	☐	For Bayesian analysis, information on the choice of priors and Markov chain Monte Carlo settings
☒	☐	For hierarchical and complex designs, identification of the appropriate level for tests and full reporting of outcomes
☒	☐	Estimates of effect sizes (e.g. Cohen's *d*, Pearson's *r*), indicating how they were calculated

Our web collection on statistics for biologists contains articles on many of the points above.

Software and code

Policy information about availability of computer code

Data collection	PET/CT imaging was performed on GNEXT (Sofie Biosciences). 3D SBEM images were acquired using Gatan 3View 2XP micritome system, and were aligned and reconstructed using IMOD imaging processing package available online. MicroCT images was acquired on a Zeiss Versa 510 microscope. Live-cell imaging was acquired on a Zeiss LSM 880 with Airyscan and image deconvolution was processing in ZEN software. Respiration analysis were performed on a Seahorse XFe96 analyzer.
Data analysis	Segmentation of individual cells from 3D SBEM images was performed in Amira with XImagePAQ extension. Mitochondrial morphology and distribution in fluorescent images were analyzed in ImageJ (FIJI, v3.3.2) with built-in function and plugins customized for this study. Mitochondrial cristae was fragmented and analyzed using Trainable Weka Segmentation built-in ImageJ (FIJI). Mitochondrial distribution in 3D SBEM images was measured by "mtk" program that is available on GitHub. Statistical analyses were performed on GraphPad Prism 9 and R studio (R 4.2.1). IHC staining was analyzed in QuPath(0.2.3)

For manuscripts utilizing custom algorithms or software that are central to the research but not yet described in published literature, software must be made available to editors and reviewers. We strongly encourage code deposition in a community repository (e.g. GitHub). See the Nature Research guidelines for submitting code & software for further information.

Data

Policy information about availability of data

All manuscripts must include a data availability statement. This statement should provide the following information, where applicable:

- Accession codes, unique identifiers, or web links for publicly available datasets
- A list of figures that have associated raw data
- A description of any restrictions on data availability

Source data for western blots are provided in Supplementary Fig.1. Data that support the findings of this study haven been deposited in CELL IMAGE LIBRARY (http://cellimagelibrary.org/groups/54862) or are available from the corresponding author upon reasonable request. Source data are provided with this paper.

Field-specific reporting

Please select the one below that is the best fit for your research. If you are not sure, read the appropriate sections before making your selection.

☒ Life sciences ☐ Behavioural & social sciences ☐ Ecological, evolutionary & environmental sciences

For a reference copy of the document with all sections, see nature.com/documents/nr-reporting-summary-flat.pdf

Life sciences study design

All studies must disclose on these points even when the disclosure is negative.

Sample size	We determined the sample size of in vivo studies on genetically modified mouse models based on the range of confidence internal for the treatment effects, our previous publications (PMID: 31666695; 29763624), and the requirements from Animal Research Committee (ARC) to minimize the number of animals involved. The sample size of in vitro cultured cells was determined by preliminary experiments with positive and negative control and statistical classifiers collected from the preliminary experiments. We determine the sample size which enables the experimental design will be sensitive to the potential difference in the treatments. No statistical methods were used to predetermine sample size.
Data exclusions	There was no data excluded in this study.
Replication	All experiments were repeated in at least duplicate. The experiments were replicated independently and all attempts at replication were successful. For in vitro cultured cells, we performed independent repeated runs with cultures of same passage number on different days. The replicate experiments were performed with the same method on the same equipments. We included technical replicates in each independent run to reduce error. We used F-test for the equality of variance to examine whether the replicates are significantly different from each other.
Randomization	Sample groups were allocated randomly. We randomized the selection of mice in terms of age, gender, weight, litter for experiments and assigned the treatments or vehicle to mice in random order.
Blinding	Image acquisition was performed by personnel different from researcher who administrated treatments and sample preparation with blind-labeling (e.g. group A, B, C ect.). Image analysis was performed by machine algorithms or automatic programming scripts. Investigators were not blinded for other data collection procedures.

Reporting for specific materials, systems and methods

We require information from authors about some types of materials, experimental systems and methods used in many studies. Here, indicate whether each material, system or method listed is relevant to your study. If you are not sure if a list item applies to your research, read the appropriate section before selecting a response.

Materials & experimental systems

n/a	Involved in the study	
☐	☒	Antibodies
☐	☒	Eukaryotic cell lines
☒	☐	Palaeontology and archaeology
☐	☒	Animals and other organisms
☒	☐	Human research participants
☒	☐	Clinical data
☒	☐	Dual use research of concern

Methods

n/a	Involved in the study	
☒	☐	ChIP-seq
☒	☐	Flow cytometry
☒	☐	MRI-based neuroimaging

Antibodies

Antibodies used	SP-C (1:5000, AB3786 Milipore); Glut1 (1:2000, GT11-A, Alpha Diagnostic); Ndufs1 (1:1000, ab169540, abcam); O-Linked N-Acetylglucosamine (1:1000, ab2739, Abcam); SDHA (1:1000, 5839, Cell Signaling Technology); SDHC (1:1000, ab155999, Abcam);

	Actin (1:5000, A3853, Sigma); Tubulin (1:2500, T9026, Sigma); CD 34 (1:800, ab8158, Abcam); TTF-1 (1:1000, M3575, Dako); Cytokeratin 5 (1:1000, ab52635, Abcam)
Validation	Antibodies used in this study have been validated on the commercial websites and referenced by previous publications. SP-C (1:5000, AB3786 Milipore), Glut1 (1:2000, GT11-A, Alpha Diagnostic), and Ndufs1 (1:1000, ab169540, abcam) were validated in our previous publications; O-Linked N-Acetylglucosamine (1:1000, ab2739, Abcam), Actin (1:5000, A3853, Sigma) and Tubulin (1:2500, T9026, Sigma) antibodies have been validated on the commercial website and cross-validated by multiple publications. SDHA (1:1000, 5839, Cell Signaling Technology), SDHC (1:1000, ab155999, Abcam); Actin (1:5000, A3853, Sigma); Tubulin (1:2500, T9026, Sigma); CD 34 (1:800, ab8158, Abcam); TTF-1 (1:1000, M3575, Dako); Cytokeratin 5 (1:1000, ab52635, Abcam).

Eukaryotic cell lines

Policy information about cell lines

Cell line source(s)	A549 cells and H1975 cells were obtained from ATCC. The lung squamous cell line (human) RH2 were established in the laboratory of Dr. Steven Dubinett (UCLA). The head and neck squamous cell line (human) Tu686 was a gift from Maie St.John laboratory (UCLA). The mouse lung squamous cell line derived from Mouse 5 and mouse lung adenocarcinoma cell line derived from Mouse 4 were established in our laboratory. A549 Rho cells were generated based on the protocols from previous publication (PMID: 18353857). Mycoplasma tested using the LookOut Mycoplasma PCR Detection Kit (Sigma).
Authentication	The identity of human NSCLC and HNSCC cell lines was confirmed by Laragen Inc. using short tandem repeat DNA analyses.
Mycoplasma contamination	All cell lines were negative for mycoplasma contamination. Mycoplasma tested using the LookOut Mycoplasma PCR Detection Kit (Sigma) and confirmed by Laragen Inc.
Commonly misidentified lines (See ICLAC register)	The study did not involve commonly misidentified cell lines.

Animals and other organisms

Policy information about studies involving animals; ARRIVE guidelines recommended for reporting animal research

Laboratory animals	We employed 5 GEMMs in this study: (1) Kras-Lox-Stop-Lox-G12D; Rosa26-Lox-Stop-Lox-Luc (Kras); Kras-Lox-Stop-Lox-G12D; Lkb1 Lox/Lox; Rosa26-Lox-Stop-Lox-Luc mice (KL); Kras-Lox-Stop-Lox-G12D; p53 Lox/Lox; Rosa26-Lox-Stop-Lox-Luc mice (KP); Kras-Lox-Stop-Lox-G12D; Lkb1 Lox/Lox; p53 Lox/Lox; Rosa26-Lox-Stop-Lox-Luc mice (KPL); Lkb1 Lox/Lox; p53 Lox/Lox; Pten Lox/Lox; Rosa26-Lox-Stop-Lox-Luc mice (LPP). Mice (1)-(4) are FVB/NJ, mice (5) are of C57BL/6J x FVB/NJ mixed background. Animals used in the study were of both sexes and 8-35 weeks. Animals were housed with 12h light/dark cycle, 30-70% humidity.
Wild animals	The study did not involve wild animals.
Field-collected samples	The study did not involve field-collected samples.
Ethics oversight	All animal experiments were approved by Animal Research Committee (ARC) at University of California Los Angeles and performed following ARC protocols and requirements. The recombinant DNA and bio-hazardous materials and procedures were approved by Institutional Biosafety Committee (IBC) at University of California Los Angeles.

Note that full information on the approval of the study protocol must also be provided in the manuscript.
